# Type 2 diabetes, gut microbiome, and systems biology: A novel perspective for a new era

**DOI:** 10.1080/19490976.2022.2111952

**Published:** 2022-08-24

**Authors:** Yoscelina Estrella Martínez-López, Diego A. Esquivel-Hernández, Jean Paul Sánchez-Castañeda, Daniel Neri-Rosario, Rodolfo Guardado-Mendoza, Osbaldo Resendis-Antonio

**Affiliations:** a Human Systems Biology Laboratory. Instituto Nacional de Medicina Genómica (INMEGEN). México City, México; b Programa de Doctorado en Ciencias Médicas, Odontológicas y de la Salud, Universidad Nacional Autónoma de México (UNAM). Ciudad de México, México; c Metabolic Research Laboratory, Department of Medicine and Nutrition. University of Guanajuato. León, Guanajuato, México; d Programa de Maestría en Ciencias Bioquímicas, Universidad Nacional Autónoma de México (UNAM). Ciudad de México, México; e Research Department, Hospital Regional de Alta Especialidad del Bajío. León, Guanajuato, México; f Coordinación de la Investigación Científica – Red de Apoyo a la Investigación, Universidad Nacional Autónoma de México (UNAM). Ciudad de México, México

**Keywords:** Gut microbiome, type 2 diabetes, diet, physical activity, anti-diabetic drugs, high-throughput data, personalized medicine, systems biology, dysbiosis

## Abstract

The association between the physio-pathological variables of type 2 diabetes (T2D) and gut microbiota composition suggests a new avenue to track the disease and improve the outcomes of pharmacological and non-pharmacological treatments. This enterprise requires new strategies to elucidate the metabolic disturbances occurring in the gut microbiome as the disease progresses. To this end, physiological knowledge and systems biology pave the way for characterizing microbiota and identifying strategies in a move toward healthy compositions. Here, we dissect the recent associations between gut microbiota and T2D. In addition, we discuss recent advances in how drugs, diet, and exercise modulate the microbiome to favor healthy stages. Finally, we present computational approaches for disentangling the metabolic activity underlying host-microbiota codependence. Altogether, we envision that the combination of physiology and computational modeling of microbiota metabolism will drive us to optimize the diagnosis and treatment of T2D patients in a personalized way.

## Introduction

Type 2 diabetes mellitus (T2D) is a metabolic disorder characterized by hyperglycemia as a result of insulin resistance (IR) and a relative lack of insulin in the human body.^1^ Notably, dysbiosis of the gut microbiome accompanies the progression of IR in T2D and the development of microvascular (retinopathy, nephropathy, and neuropathy) and macrovascular (atherosclerosis) complications of diabetes.^2,3^ This dysbiosis remodels the intestinal barrier and insulin signals through metabolites derived from bacteria, which interact with receptors on epithelial, fat, muscle, liver, pancreatic, and cardiac cells. Thus, metabolic signals produced by the gut microbiome can indirectly promote IR by altering the host’s metabolism. Among these metabolic changes, we highlight metabolic endotoxemia and the low rate of production of short-chain fatty acids (SCFAs) and secondary bile acids (BAs).^1^ Although these findings have helped to characterize the association between the gut microbiome profile and T2D, it remains a challenge to grasp the mechanisms that drive their codependence; it is also difficult to use this knowledge to modulate the metabolic crosstalk between the microbiome and host.^4^

Currently, there is great interest in controlling external factors to modulate host-microbiome metabolic crosstalk and to restore patients to a healthy state. Thus, classical variables associated with lifestyle, such as diet and exercise, have increased their relevance in personalized medicine.^5^ Moreover, new technologies, such as fecal microbiota transplantation or bacteriophage intervention (phagosome), are promising technologies for enhancing patients’ wellness and treatment.^6,7^ Currently, two approaches are helping to reach these objectives. On the one hand, 16S rRNA and shotgun metagenome technologies allow us to carefully monitor the physiological state and composition of the gut microbiome in patients with different degrees of T2D.^8,9^ This massive amount of biological data contributes to characterizing the phenotype state of patients and evaluating how these phenotypes are altered as the disease progresses. On the other hand, the development of computational modeling approaches (CMAs) with the capacity to integrate high-throughput (HT) data is a pioneering effort to elucidate the ecological interactions of the bacterial community, and to postulate the metabolic mechanism by which T2D progresses. In particular, computational models based on inference microbial interactions and genome-scale metabolic reconstructions have emerged as a remarkable scheme required to understand the metabolic activity of the gut microbiome and to track the changes that follow the emergence of T2D.^9^ Currently, this field is nascent, but some pioneering advances have been reported in T2D and type 1 diabetes (T1D). In this narrative review, we analyze the state of the art of the association between the gut microbiome and T2D, the factors that modulate the interaction, and some in silico strategies to reveal the underlying metabolic mechanisms. First, we discuss the cutting-edge evidence of the relationship between host-microbiota metabolism and physiological alterations associated with T2D. Then, we discuss recent publications that highlight the importance of handling microbiome composition through lifestyle, diet, and promising intervention methods to modulate the gut microbiome. Finally, the last section is devoted to presenting and discussing frontiers in computational strategies to describe the complex interactions in the bacterial community, shed light on their organization, and build testable hypotheses to modulate metabolic mechanisms. Overall, our review highlights the importance of combining physiological knowledge, HT technologies, and computational modeling of microbiome metabolisms for designing microbiome interventions in favor of a healthy phenotype. To add an original contribution on the state of the art, the structure of the review was grounded in a bibliometric analysis with the Bibliometrix R library.^10^ This analysis was built with the terms “type 2 diabetes”, “gut microbiota”, “gut microbiome”, “systems biology” and “bioinformatics”, as keywords to find either in the documents’ titles or abstracts. Next, we filtered this search based on the review article as a type of document. Interestingly, we did not detect any document related to the content of our manuscript (Table S1-S2). Undoubtedly, the achievements around this field will have a strong impact on precision medicine for optimizing the outcome of treatments and improving patients’ quality of life.

## The gut microbiome and T2D

The gut microbiome is a complex microbial ecosystem that coexists with various biological processes and metabolic capacities in the host.^11^ Through interactive evolutionary processes, hosts and their microbiomes have established mutual benefits. With the abundant evidence of this relationship and its influence on health, humans and their gut microbiome can be considered holobionts, and the health of the host depends on the microbiota and cannot be seen as disconnected from it.^12^ Nevertheless, there are intrinsic (genetics, age, sex, and health condition) and environmental factors (diet, antibiotic consumption, and lifestyle) that affect the composition of the gut microbiome and its structural functions. These factors, known as microbial disruptors, can alter a variety of physiological mechanisms that favor the development of pathologies such as intestinal permeability, chronic low-grade inflammation, and changes in carbohydrates metabolism and its associated signaling pathways (the insulin route).^13^

**Figure 1.**
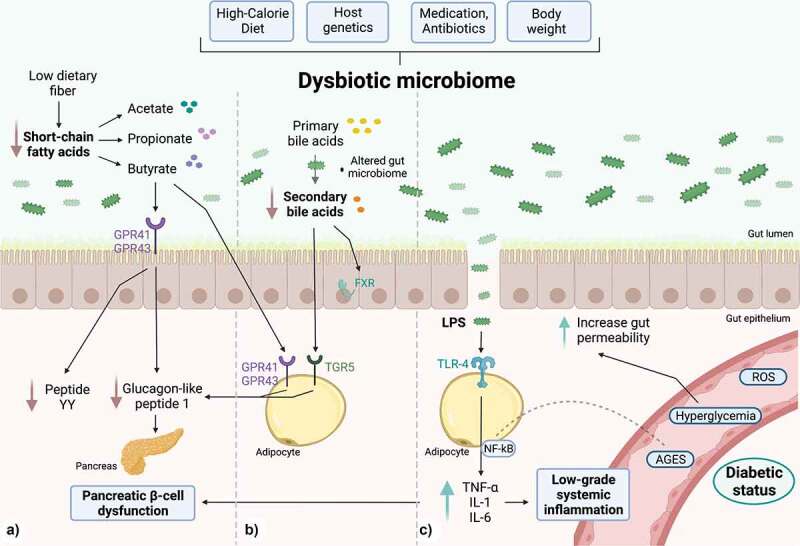
Gut microbiome-derived mechanisms are able to modulate the chronic inflammatory state in DT2. a) SCFAs, products of dietary fiber fermentation, promote GLP-1 and YY peptide secretion in L cells by activating G protein-coupled receptors such as GPR41 and GPR43. With a dysbiotic microbiome, there is an overall decline in the production of SCFAs, leading to a reduction in GLP-1 secretion, impairing pancreatic insulin secretion. b) Secondary bile acids derived from the intestinal microbiome act as mediator molecules through nuclear receptors such as the FXR receptor and the TGR5 membrane receptor, which in intestinal L cells improve glucose metabolism by stimulating GLP-1 production and promoting insulin secretion. Also, in muscle (not shown), they enhance mitochondrial activity and facilitate insulin sensitization. c) PAMPs, e.g., LPS can bind to the TLR4 receptor, and stimulate the expression of pro-inflammatory cytokines IL-6, IL-1 and TNF-α, which are characteristic of a low-grade systemic inflammatory state. There is an increase in intestinal permeability due to the direct effects of glucotoxicity and gut dysbiosis. Created with BioRender.com.

Understanding the biochemical processes related to the interaction between microbial disruptors and the gut microbiome could explain the remarkable relationship of the gut microbiome with its hosts. From a metabolic point of view, the gut microbiome can be conceived as a bioreactor inside the host, leading to the production of bioactive compounds, and whose dysbiosis could be associated with the development of T2D.^14^ The set of metabolites derived from the gut microbiome serves as a source of signals that facilitate communication between the body’s organs via the nervous system (afferent and efferent autonomic pathways). Through these signals, the gut microbiota (GM) modulate the immune, endocrine, gastrointestinal, and nervous systems, forming the microbiota-gut-brain axis.^15^ When bacteria-host communication fails, vital functions of the host are interrupted, causing numerous dysfunctions associated with disease. In the case of T2D, several studies have characterized the composition of GM and have confirmed the existence of a particular dysbiosis depending on biogeographical variables.^16,17^ For instance, in European and Chinese populations, remarkable differences between T2D and healthy subjects were the low relative abundances of butyrate-producing bacteria (*Roseburia intestinalis* and *Faecalibacterium prausnitzii*) and the higher relative abundance of species such as *Lactobacillus*, as well as some opportunistic pathogens like *Bacteroides caccae, Clostridium hathewayi, Clostridium ramosum, Clostridium symbiosum*, and *Escherichia coli*.^18,19^ Previous results suggest that an interesting aspect of these findings is that GM has been associated with the immune system (IS) through immunoinflammatory signaling, which can affect insulin sensitivity (Figure 1).^20^

Of the most evident associations between T2D and physiological alterations, we highlight those comprising the gut microbiome and alterations of glucose metabolism through SCFA and BA secondary reduction (Figures 1A and 1b).^14,21^ GM typically generate SCFAs through the saccharolytic fermentation of dietary fiber. SCFA production is carried out mainly in the distal and proximal colon at a 60:20:20 molar ratio (acetate, propionate, and butyrate).^22^ Fifteen grams of non-digestible carbohydrates in the colon subjected to saccharolytic fermentation produce between 400 and 600 mmol/day of SCFAs.^23^ The effectiveness of this biochemical transformation depends on numerous factors, including pH, which influences the growing competition of bacteria in the intestine. Bacteria such as *Lactobacilli, Roseburia, Faecalibacterium prausnitzii*, and *Bifidobacteria* promote health in the host and enhance the production of SCFAs at a pH of 5.5. Alternatively, when the non-fermentable fiber is limited in the distal large intestine, the luminal pH is raised to 6.5, eliminating butyrate-producing bacteria almost entirely.^24^

GM uses major bacterial metabolic pathways (Embden-Meyerhof-Parnas, pentoses-phosphate, Wood-Ljungdahl, succinate, acrylate, and propanediol) to produce SCFAs.^25^ SCFAs play a role in insulin sensitivity in humans through incretins, which are intestinal peptides that act as hormones produced in the gastrointestinal tract by enteroendocrine cells in response to food intake. At a functional level, incretins affect the cells of the islets of Langerhans by increasing the secretion of insulin by 70% in the postprandial state and consequently decreasing glucagon secretion depending on circulating glucose. The main incretins are glucagon-like peptide 1 (GLP-1) and glucose-dependent insulinotropic peptide (GIP). SCFAs, products of saccharolytic fermentation, bind to G protein-coupled free fatty acid receptors in the colon mucosa, which are G-protein-coupled receptor type 41 (GPR41) and G-protein-coupled receptor type 43 (GPR43). Through the GPR41 receptor, the YY peptide is released, which reduces the energy extracted from the diet, increasing intestinal peristalsis. In contrast, GPR43 induces glucose-dependent insulin discharge, inhibits glucagon secretion, improves insulin sensitivity, reduces hepatic gluconeogenesis, and controls appetite (Figure 1a).^26^ Furthermore, SCFAs can prevent obesity and IR by shifting from lipogenesis to fatty acid oxidation in the liver and adipose tissue. There is evidence supported in a murine model fed a high-fat diet, where butyrate supplementation prevents an increase in body weight and increases insulin sensitivity.^27^ In addition, butyrate and propionate can induce intestinal gluconeogenesis, acting through a gut-brain neural circuit to improve peripheral glucose production and insulin sensitivity.^28^

On the other hand, several studies have reported changes in the concentration of circulating bile acid groups (BAs) in subjects with IR or T2D.^29,30^ These alterations were related to liver structures (primary BAs) and derivatives produced by intestinal microbes (secondary BAs).^31^ BAs are signaling molecules that regulate glucose and lipid metabolism. The primary BAs are secreted in the small intestine after production and glyco-taurus conjugation (N-acyl amidation with glycine or taurine substituents) in the liver. Through a bacterial hydrolase enzyme (produced by *Clostridium, Bacteroides, Lactobacillus, Bifidobacterium*, and *Enterococcus*), deconjugation is first generated.^32^ There is evidence pointing out that 95% of conjugated primary BAs are reabsorbed through enterohepatic circulation, while the remaining 5% escape from this mechanism, reaching the large intestine and becoming secondary BAs by the action of *Firmicutes* (*Eubacterium spp* and *Clostridium spp*).^33^

Subjects with T2D have a lower number of secondary BAs than healthy subjects. This characteristic is related to an alteration in carbohydrate metabolism since they have an insulin-sensitizing role.Thus, BAs have been proposed as metabolic integrators of energy homeostasis involved in the regulation of numerous metabolic pathways, including their synthesis and enterohepatic circulation.^31^ This regulation of energy metabolism would be produced through nuclear receptors, identical to the farnesoid X receptor (FXR) and the G-protein-coupled bile acid receptor (Gpbar1). Specifically, Takeda G-protein–coupled receptor 5 (TGR5) is a bile acid membrane receptor expressed in the gallbladder, ileum, colon, and brown and white adipose tissue.^34^ This receptor internalizes and activates a series of adenylate cyclase-dependent signals through the involvement of glucose metabolism and lipid energy in brown adipose tissue and muscle, heightened mitochondrial activity, and phosphorylation. These processes stimulate insulin sensitization in murine models of diabetic and obese conditions. Likewise, in L cells, TGR5 improves glycemic metabolism by stimulating GLP-1 production and insulin secretion (Figure 1b).^33^ Furthermore, secondary BAs appear to have an insulin-sensitizing role. These act as mediator molecules through nuclear receptors such as FXR receptor and the TGR5 membrane receptor expressed in myriad tissues, such as the vesicle, ileum, colon, brown adipose tissue (BAT), and white adipose tissue (WAT). In BAT and muscle, increased mitochondrial activity and phosphorylation lead to insulin sensitization in obese and diabetic mouse models.

Instead, innate immune cells (monocytes and macrophages) are considered key players in the pathogenesis of T2D, as they can recognize microbial signals such as LPS and peptidoglycan, which are considered PAMPs. In addition, they have the ability to recognize non-microbial signals released by cellular damage or stress, such as mitochondrial DNA and ATP, which are considered DAMPs.^35^ The signals from the gut microbiome regulate innate immunity and influence local and systemic responses.^36,37^ In T2D, microbiome signals can promote low-grade chronic inflammation associated with IR through toll-like receptors (TLRs). Cell surface TLRs mainly recognize microbial membrane components such as lipids, lipoproteins, and proteins. Specifically, TLR4 recognizes bacterial lipopolysaccharide (LPS).^38^ TLRs differentially recruit members of a set of TIR domain-containing adaptors, such as MyD88, which is utilized by all TLRs (except TLR3) and activates nuclear factor kappa-light-chain-enhancer of activated β-cells (NF-κB) and mitogen-activated protein kinases (MAPKs) for the induction of inflammatory cytokine genes.^36,37^ Due to a high-fat diet, these receptors are activated in response to microbial stimuli such as LPS, which is present on the membranes of gram-negative bacteria.^39^ TLRs, specifically TLR-4, also interact with glycosylated serum proteins called advanced glycosylation end products (AGEs)^40,41^ to activate the transcription of MAPKs and NF-κB. In turn, this last pathway acts as an enhancer of activated β-cells after activation by LPS.^39^ On the other hand, proteins (such as hemoglobin, albumin, and low-density lipoproteins [LDL]) are enzymatically glycosylated in lysine and arginine residues in the hyperglycemic state. As a consequence of this glycosylation, the production of AGEs is favored.^40,41^ AGEs in macrophages can bind to TLR-4, which is overexpressed in T2D. Thus, TLR activation leads to a cascade of intracellular signaling mediated by NF-κB when it is translocated to the nucleus, and activates the transcription of genes coding for cytokines and inflammatory chemokines, including tumor necrosis factor-alpha (TNF-α), interleukin 1 beta (IL-1β), and interleukin 8 (CXCL8). As a result, this process promotes the onset of the inflammatory response to hyperglycemia (Figure 1c).^42^

Under normal physiological conditions, insulin binds to the receptor located on the surface of myocytes, hepatocytes, and adipocytes.^43^ Subsequently, an intracellular signaling cascade mediated by the insulin receptor substrate (IRS) is initiated, which activates protein kinase B (Akt), and finally induces the mobilization of two isoforms of glucose transporter (GLUT-2 and GLUT-4) to introduce glucose into the cell. When β-cells are subjected to an inflammatory process, they respond through the activation of two main pathways: 1) the activation of TLR2 and TLR4 by PAMPs or DAMPs; and 2) the assembly of the inflammasome nucleotide-binding oligomerization domain (NOD)-like receptor pyrin domain-containing (NLRP3).^44^ This inflammatory process causes insulitis, characterized by a continuous release of IL-6, IL-8, TNF-α, and monocyte chemotactic protein 1 (MCP-1), and by the activation of insular macrophages and the recruitment of new monocytes and peripheral macrophages.^43^ When the inflammatory response is improperly regulated, it produces a state of damage called chronic low-grade inflammation.^36^ During low-grade systemic inflammation, adipose, liver, and muscle tissue endure infiltration of inflammatory macrophages producing TNF-α; this cytokine directly interferes with the tissues and reduces their ability to respond to insulin. TNF-α reduces tyrosine phosphorylation of the insulin receptor and IRS-1, and induces serine phosphorylation of IRS1, which in conjunction reduces downstream transduction of insulin signaling.^45^ It has recently been postulated that the presence of gut dysbiosis favors an increase in the prolonged circulation of inflammatory markers and increased intestinal permeability.^46^ In turn, bacterial products (PAMPs) enter the circulatory system and promote the genesis of systemic inflammation in T2D.^42^ Overall, the genesis of T2D is conditioned by high levels of pro-inflammatory cytokines and the composition of the GM.^47,48^

Given their importance, in the following sections, we describe the relationship among the gut microbiome and two variables with relevance in T2D physiopathology: IR and pancreatic β-cell dysfunction (PBD).

### Insulin resistance

**Figure 2.**
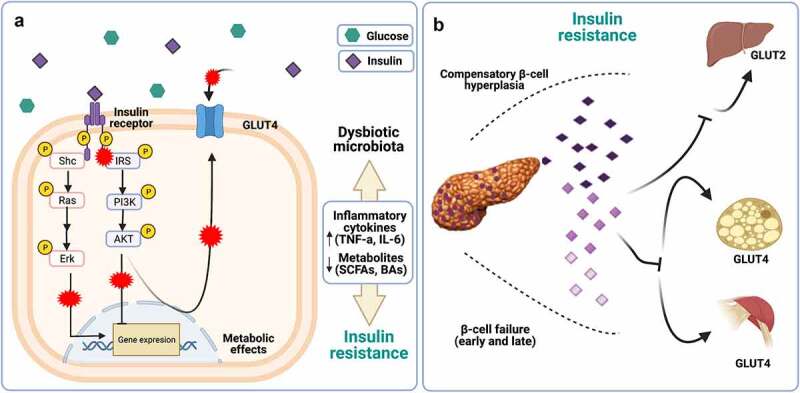
Defects in glucose transport in IR. a) Increased expression of inflammatory cytokines (TNF-a, IL −1, IL6) dependent on TLR4/MYD88 activation are negatively involved in downstream insulin signaling (marked with red crosses). Furthermore, an imbalance in the production of microbiota-derived metabolites, including SCFAs and BAs, is indirectly related to insulin resistance through the modulation of their receptors. b) Hyperglycemia is a consequence of over-demand of insulin requirements; indeed, β-cells improve the restoration of glucose homeostasis through increased insulin biosynthesis. Over time, apoptosis exceeded the rate of replication, resulting in loss of β-cells and a reduction in β-cell mass. Created with BioRender.com.

The gut microbiome plays an important role in the progression of IR to T2D.^20^ An imbalance between potentially pathogenic and nonpathogenic bacteria can induce crucial metabolic alterations in the entire organism, which in turn can disturb the physiological parameters in multiple body compartments.^49^ This perturbation has at least two complex consequences. First, IR promotes alterations in different metabolic pathways, such as lipids, amino acids, and bile acids. Second, these alterations have substantial implications for the modulation of insulin sensitivity.^14^ In this way, metabolites produced by GM may regulate insulin sensitivity through several components of the insulin signaling pathway, such as insulin receptor substrates (IRS) and the enzyme kinase AKT (Figure 2a).^50^ Furthermore, some of these IRSs can indirectly affect the flow of substrates through lipogenesis, lipid oxidation, protein synthesis and degradation, and hepatic gluconeogenesis.

On the other hand, branched-chain amino acids (BCAAs) have recently attracted significant scientific attention because elevated blood concentrations of BCAAs are associated with states of IR, such as obesity and T2D.^51^ The main species associated with T2D and BCAA biosynthesis are *Prevotella copri* and *Bacteroides vulgatus* in the Danish population.^52^ These bacteria decreased the expression of BCAAs through catabolizing enzymes in adipose tissue and liver, and promoted an increase in inflammation in WAT, stress on the endoplasmic reticulum (ER), and an alteration in insulin signaling at the hypothalamic level.^14^ Additionally, BCAAs increase lipid oxidation in muscle, accumulating acylcarnitines and generating mitochondrial dysfunction. In adipose tissue, the alteration of BCAA catabolism reduces substrate flow to lipogenesis, contributing to metabolic dysfunction in IR and linking it to the progressive loss of pancreatic β-cell function.^14^ Ultimately, these changes generate an acute secretory response in the β-cell, resulting in a progressive dysfunction characterized by an abnormal secretory process due to the pancreatic β-cell trying to adapt and compensate for the IR, as described in the following section.

### Pancreatic β-cell dysfunction

Glucose homeostasis is the product of metabolic, hormonal, neural, and microbial signals whose regulation determines the degree of glucose-dependent insulin release. The progression from normoglycemia to glucose intolerance and later to T2D occurs due to a deterioration in these signals, which gradually decreases insulin sensitivity and β-cell functionality (Figure 2b).^2^ This dysfunction associated with GM can be induced by two effects: 1) the increase in intestinal permeability; and 2) the chronically elevated glucose levels in the host (islet glucotoxicity).^53^ This physiological imbalance causes an increase in intestinal permeability, which in turn promotes the translocation of some bacteria (*Proteus mirabilis* and *Escherichia coli)* and their metabolites.^54^ To respond to intestinal permeability in the host, two main detection systems continuously scan for bacteria capable of translocating the intestinal mucosa or adhering to the epithelium: 1) NOD-like receptors (NLRs), which detect the presence of intracellular microbes; and 2) TLRs.^38^

The first detection system involves NLRs, specifically the NLR family pyrin domain containing (NLRP) subfamily, which can oligomerize into a macromolecular complex known as an inflammasome. Inflammasomes are found in the cytosol and mediate the activation of inflammatory caspases.^55^ Activation of the inflammasome depends on two pathways: first, NF-κB expression upon sensing PAMPs or DAMPs by their respective receptors, e.g., TLR4 binds to LPS. This process alerts the cell to express inflammasome-related genes such as inactive NLRP3, Pro-IL1, and Pro-IL-18. The second signal involves the recognition of bacterial toxins (e.g., cytosolic bacterial peptidoglycan) or host metabolites (potassium efflux) by inflammasome sensor proteins (NLRP3, NLR Family CARD Domain Containing 4 [NLRC4]).^56^ This recognition system allows recruitment of the adaptor protein ASC, which binds to pro-caspase-1, leading to its self-processing and activation. Caspase-1 cleaves the inactive precursor proteins of IL-1β and IL-18 into their bioactive fragments. In addition, this protease induces an inflammatory cell death known as pyroptosis.^53,55^ The second detection system for bacterial permeability corresponds to TLRs. These are pattern recognition receptors (PRRs) capable of recognizing PAMPs and inducing the expression of pro-inflammatory cytokines (IL-1B, IL-6, and TNF-α), mediated by the adapter molecules MyD88 and NF-κB. Taken together, these mechanisms directly affect the function of β-cells.^53^ Spranger et al. reported that the combined elevation of IL-6 and IL-1β, products of the activation of the two detection systems, was related to a threefold increased risk of developing T2D.^57^

Concerning islet glucotoxicity, chronically elevated glucose levels have been reported to impair islet function and proliferation, and induce apoptosis. In addition, the intra-islet expression of IL-1β may contribute to T2D pathogenesis by inducing the loss of mass and β-cell function. Consequently, hyperglycemia and glucotoxicity result from pancreatic dysfunction, increase intestinal permeability, and activate the inflammatory response.^53,58^

## Factors that modulate the microbiome in patients with T2D

**Figure 3.**
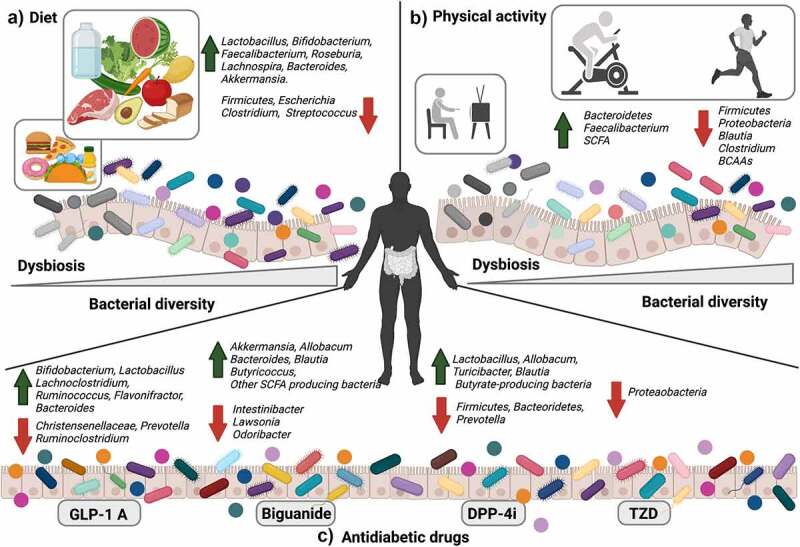
Factors that modulate the microbiome. a) Diet: The correct diet modifies the structure, and GM function increases bacterial diversity and SCFA producing species that contribute to better glycemic control in T2D patients. b) Physical activity: In sedentary subjects with T2D, physical activity increases bacterial diversity and SCFA-producing species. These changes reduce endotoxemia and increase the degradation of SCFAs and BCAAs. c) Antidiabetic drugs change the composition, diversity, and SCFA-producing bacteria in the GM, favoring glucose homeostasis through their mechanisms of action. Created with BioRender.com.

GM have great plasticity, which implies the ability to adapt their populations to fluctuating environmental conditions.^59^ Currently, their composition and diversity are modulated through classical factors such as diet, exercise, drugs, and more recently through interventions such as fecal microbiota transplantation (FMT), bacteriophage intervention through phageome studies, antibiotics, and bariatric surgery. All of these modulator methods can generate beneficial changes in the structure and function of the GM and restore them temporarily or permanently.^60^ In the next section, we will analyze the effect of these modulators on microbiome composition (Figure 3).

### Diet

Treatment strategies for T2D include lifestyle changes, such as dietary interventions with routine physical exercise.^61^ Diet is a fundamental environmental contributor that interferes with the structure and function of the GM, playing a key role in modulating the benefits of human health.^60^ Studies on diet and microbiome associations have shown that the consumption of prebiotics and dietary fibers increases the abundance of SCFA-producing bacteria in the gut.^62^ At present, this modulator has been extended to include dietary patterns, eating behaviors, diet quality, and food preparation methods.^63^ Therefore, an unbalanced diet causes unfavorable changes in energy balance and GM composition. This dysbiosis results in weight gain and a higher risk of developing metabolic illnesses. On the other hand, a balanced diet favors appropriate changes in the microbiome composition and can promote weight loss and a healthy metabolic transformation.^16^ Therefore, nutrient ratios in meals affect digestive secretions, absorption, and transit time. These factors also affect human GM.^63^ Recent studies have estimated that for an average diet, 40 g of carbohydrates, 12 to 18 g of protein, and 2 to 10 g of fat reach the colon undigested every day and serve as substrates for microbial metabolism.^64^ Based on recent estimations, the gut microbiome contains 16,000 active carbohydrate enzymes and 9,000 genes involved in the complex metabolism of carbohydrates and polysaccharides, while the human genome contains only 17 of these genes.^63^ The superiority of gut microbes to metabolize a wide range of non-digestible nutrients is remarkable. In this way, dietary interventions modulate the GM, and these changes might contribute to better glycemic control in T2D patients.^65^

Advances in microbiome research have revealed the importance of diet variability and its contribution to GM composition in supporting health. Because diet influences intestinal transit time, pH, and macronutrient intake, possible approaches to achieving a healthy microbiota are to directly manage beneficial bacteria through probiotics, a Mediterranean diet, diet restrictions, a content-modified diet, and a diet high in non-digestible polysaccharides.^66^ The shift in diet has a dynamic effect on the composition of the GM in the short and long term and is dependent on the type of food eaten.^67^ Table 1 summarizes studies with statistically significant differences in the composition after dietary intervention for T2D patients.^68–70^ Furthermore, the differences in clinical parameters are closely related to changes at various levels of taxa (phylum, family, genus, species, and *Firmicutes: Bacteroidetes* relationship) (Table 1 and Table S3).

**Table 1.** Descriptive characteristics and assessment of diet, physical activity, and antidiabetic drugs in GM of T2D patients.

Nutritional/dietary interventions								
Country year	Participants	Sample size	Intervention implemented	Treatment duration	Aims/Outcomes	Gut-microbiota significant differences	Clinical effects	Ref
Portugal 2021	Male and female. 40–80 years old. T2D.	9	**Intervention**: The Mediterranean diet.	12 weeks	**Primary**: This study was to evaluate the modulation role of the Mediterranean diet in gut microbiota.	The effect size induced by the Mediterranean diet on the *Firmicutes* to *Bacteroidetes* ratio was considered clinically relevant after 12 weeks (p = .846; Cohen d = −0.83). *Prevotella* to *Bacteroides* ratio tended to increase right after 4 weeks of the intervention (*p* = .438; 4 weeks Cohen d = 0.74 and 12 weeks Cohen d = 0.74).	Twelve weeks after the intervention, HbA1c decreased by 0.67% (7.53 ± 1.07% to 6.86 ± 0.85%, *p* < .05; Cohen d = −0.70) and HOMA-IR decreased from 3.79 ± 2.98 to 2.76 ± 2.05 (*p* < .05; Cohen d = −0.41), with no accompanying changes in lipid profile.	68
China 2020	Male and female. ≥ 18 years old. T2D.	45	**Intervention**: a-LCD + 84 g/day almond. **Control**: LFD + 56 g/day almond.	3 months	**Primary**: This study was to determine the effect of an a-LCD on depression and glycometabolism. **Secondary**: This study was to determine the effect in the gut microbiota and fasting GLP-1 in patients with T2D.	At the phylum level, *Firmicutes* in the a-LCD group was significantly lower than that in the LFD group by the third month. At the genus level, *Roseburia* and *Ruminococcus* in the a-LCD group were significantly higher than those in the LFD group by the third month; compared to the baseline: *Eubacterium, Roseburia* increased significantly and *Bacteroides* decreased significantly in the a-LCD group.	Compared to the baseline, HbA1c levels in both groups decreased significantly (*p* < .01, *p* < .05) during the study period. At the third month, the HbA1c level in the a-LCD group decreased more than that in the LFD group (*p* < .01).	69
Iran 2020	Male and Female. Prediabetes.	120	**Intervention**: Consume 6 g/d multispecies probiotic or inulin-based symbiotic. **Control**: Consume 6 g/d of either a placebo containing maltodextrin.	6 months	**Primary**: This study was to determine the effects of probiotics and symbiotic supplementation on intestinal microbiome modifying in adults with prediabetes.	Six months’ supplementation with probiotics resulted in a statistically significant increase in abundance of *Bacteroides fragilis* to *E. coli* ratio. In the probiotic group, the relative proportion of *Firmicutes* to *Bacteroidetes* repressive was decreased significantly.	They do not report clinical parameters.	70
**Physical exercise**								
China 2020	Male and female. 40–46.5 years old. BMI between 27–30 Kg/m^2^. T2D.	14	**Intervention**: High-intensity exercise training.	12 weeks	**Primary**: This study was to determine the efficacy of high-intensity exercise in the prevention of diabetes. **Secondary**: This study was to determine the change in the gut microbiota through the intervention of exercise in all participants.	The relative abundances of 6 species, belonging to *Firmicutes, Bacteroidetes*, and *Proteobacteria*, respectively, were significantly altered after exercise. Species falling into the *Bacteroides* genus and *Clostridiales* order, most of which are involved in the production of SCFAs, underwent a significant strain-level genomic variation by exercise.	The responders showed a remarkable 42.70% and 49.60% decrease in fasting insulin and HOMA-IR index, respectively, as well as a striking 116.29% increase of Matsuda index (a comprehensive evaluation of both hepatic and peripheral insulin sensitivity derived from oral glucose tolerance test.	71
Finland 2020	Male and female. 45–53 years old. BMI between 27.5–33.5 Kg/m^2^.	49	**Intervention 1**: SIT (session consisted of 30-s exercise bouts). **Intervention 2**: MICT (40 to 60 min of moderate-intensity (60% of V˙O_2peak_ intensity).	2 weeks	**Primary**: This study was to determine the effects of SIT and MICTn intestinal metabolism and microbiota in subjects with insulin resistance.	Both training modes decreased the ratio of *Firmicutes*/*Bacteroidetes* (time, *P* = .04), and the abundance of *Blautia* spp. (time *P* = .051) and *Clostridium* spp. (time *P* = .04). Mainly due to the increase in the relative abundance of *Bacteroidetes* phyla (time, *P* = .03). *Lachnospira* genus was present in higher abundance after SIT compared with baseline (*P* = .025) and significantly higher abundance of *Veillonella* genus. Interestingly, the abundance of the *Faecalibacterium genus (F. prausnitzii)* was increased after MICT (*P* = .057) while no change after SIT was found.	Improved HBA1c (P = .003). Both training modes significantly reduced systemic inflammatory marker TNF α (p = .03) and tended to reduce C-reactive protein (p = .08) and intestinal inflammatory marker lipopolysaccharide-binding protein (p = .02). Lipopolysaccharide-binding protein correlated positively with HBA1c (r = 0.54; P = .02).	73
Italy 2019	Male and female. T2D.	30	**Intervention**: Program of endurance, resistance, and flexibility training.	6 months	**Primary**: This study was to evaluate the role of chronic exercise on gut flora composition and leaky gut in T2D stable patients.	Chronic exercise modified the composition of the gut microbiota, reduced intestinal mycelial overgrowth, leaky gut, and systemic inflammation.	They do not report clinical parameters.	76
Canada 2015	Six-week-old male type 2 diabetic db/db (C57BL/KsJ-leprdb/leprdb) and db/+ (heterozygote; control) littermates.	19	**Intervention**: Exercise (Exercise consisted of a low-intensity treadmill running 5 days/week for 60 min /session. **Control**: Sedentary.	6 weeks	**Primary**: The purpose of this study was to determine if exercise influences the gut microbial profile in normal and diabetic mice.	This study revealed a main effect of exercise, with a greater abundance of select *Firmicute*s species and lower *Bacteroides/Prevotella spp*. in both normal and diabetic exercised mice compared to sedentary counterparts. Conversely, *Bifidobacterium spp*. was greater in exercised normal but not diabetic mice (exercise for diabetes interaction).	The diabetic mice had higher blood glucose (P < .001). However, there was a trend toward an interaction between diabetic state and exercise training (P = .070), such that glucose was higher in Ex-db/+ than Sed-db/+ (12.9 ± 1.9 vs. 8.1 ± 0.6 mmol/L), but similar in Ex-db/db compared to Sed-db/db (20.4 ± 2.4 vs. 21.9 ± 1.7 mmol/L).	77
**Antidiabetic drugs**								
**Biguanide (metformin)**								
Colombia 2017	Male and female. 18–62 years old. BMI ≥18,5 kg/m^2^. T2D.	98	**Intervention**: Metformin. **Control**: Subjects without T2D.	5 months	**Primary**: This study determines the influence of metformin on the association of T2D and gut dysbiosis in an adult population.	Compared with participants without T2D, participants with diabetes taking metformin had a higher relative abundance of *Akkermansia muciniphila*.	Compared with ND participants, T2D-met+ participants had higher fasting glucose, HbA1c, and insulin resistance than ND participants and lower levels of the insulin-sensitizing hormone adiponectin (*P* < .05).	85
Sweden 2017	Male and female. 50–58 years old. T2D.	40	**Intervention**: Metformin. **Control**: Placebo.	4 months	**Primary**: This study was to determine the effects of metformin in treatment-naïve adults with DM2.	Increased abundance of *Akkermansia muciniphila* in individuals who received metformin for 4 months. *Bifidobacterium adolescentis*, fecal propionate, and butyrate concentrations were significantly increased by the metformin group.	Significant decreases in % hemoglobin A1c (HbA1c) and fasting blood glucose were observed only in the group randomized to metformin treatment.	86
Korea 2017	Five-week-old male C57BL/6 mice.	18	**Intervention**: HFD + metformin. **Control**: HFD and regular diet.	16 weeks	**Primary**: This study was to investigate the effect of metformin on the gut microbiota in elderly mice.	The abundance of the genera *Akkermansia, Bacteroides, Butyricimonas*, and *Parabacteroides* was significantly increased by metformin in mice fed an HFD.	Metformin administration for 16 weeks to mice fed an HFD significantly decreased the serum glucose level compared to mice fed only an HFD. Metformin also significantly improved glucose tolerance.	87
China 2019	Male and female. 40–75 years old. T2D.	180	**Intervention**: Antidiabetic drugs (Metformin, insulin, α- glucosidase inhibitor). **Control**: Non-therapeutic and health subject.	3 months	**Primary**: This study was to explore the interaction between the gut microbiome and T2D or hypoglycemics in the Chinese population.	Metformin increased the abundance of Spirochete, *Turicibacter, Fusobacterium*, and *Ruminococcus* (butyrate-producing bacteria), *Ruminococcus* was related to the impaired glucose control with regard to T2D.	They do not report clinical parameters.	88
**GLP-1 analogue**								
China 2017	Five-week-old male Sprague-Dawley.	60	**Intervention**: Liraglutide 0.2 g/kg and Liraglutide 0.4 g/kg. **Control**: Normal and diabetic conditions.	12 weeks	**Primary**: This study was to determine the influence of liraglutide on fecal microbiota in diabetic male rats.	SCFA-producing bacterias including *Bacteroides, Lachnospiraceae*, and probiotic bacteria, *Bifidobacterium*, were selectively enhanced in liraglutide-treated diabetic male rats. *Lactobacillus* was negatively correlated with fasting blood glucose.	Liraglutide significantly decreased serum insulin level, HOMA-IR, and IL-6 (*P* < .01).	90
China 2016	10 weeks old male ApoE -/- mice with a C57BL/6 genetic background.	60	Intervention: Hyperglycemia + liraglutide and Hyperglycemia + saxagliptin. Control: Normal glucose control, Normal glucose + liraglutide, and normal glucose + saxagliptin.	8 weeks	Primary: This study determines the structural modulation of the gut microbiota and the relationship with body weight, compared evaluation of liraglutide and saxagliptin treatment.	The enriched phylotypes were the genera *Allobaculum* and *Turicibacter* within the family *Erysipelotrichaceae*, the genera *Anaerostipes*, and *Blautia* within the family *Lachnospiraceae*, the genus *Lactobacillus* within the family *Lactobacillaceae*, genus *Butyricimonas* within the family *Porphyromonadaceae*, and the genus *Desulfovibrio* (phylum *Proteobacteria*, class *Deltaproteobacteria*).	The mean blood glucose level was significantly lower in liraglutide-treated mice compared with the control mice, who were fed ad libitum (6.70 ± 0.43 mmol/L vs. 7.62 ± 0.68 mmol/L, p = 9.0e-6). There were no substantial differences in the LPS concentrations.	96
**Dipeptidyl peptidase-4 inhibitor (DPP-4i)**								
China 2016	Four-week-old male Sprague-Dawley (SD) rats (induce TD2).	15	**Intervention**: Sitagliptin.	4 weeks	**Primary**: This study was on determining the effects of sitagliptin in rats (induce T2D).	At the level of genus, SCFA-producing bacteria, *Blautia, Roseburia*, and *Clostridium*, and probiotics *Lactobacillus, Bifidobacterium*, and so forth were identified as significantly different (T2D vs T2D-sitagliptin conditions).	Sitagliptin resulted in a significant reduction in blood glucose (p < .05).	94
China 2017	Five-week-old male Sprague-Dawley SD rats.	30	**Intervention**: Vildagliptin 0.01 g/kg + HFD and Vildagliptin 0.02 g/kg + HFD. **Control**: Control, control + Vildagliptin 0.02 g/kg and HFD + STZ.	12	**Primary**: This study was to identify whether vildagliptin modifies the gut microbiota structure during T2D treatment.	At the phylum level, a higher relative abundance of *Bacteroidetes*, lower abundance of *Firmicutes*, and reduced ratio of *Firmicutes*/*Bacteroidetes* were observed in the vildagliptin-treated group. Moreover, vildagliptin treatment increased butyrate-producing bacteria, including *Bacteroides* and *Erysipelotrichaeae*, in the diabetic rats.	Both doses of vildagliptin treatment reduced the fasting blood glucose and HbA1c levels (P < .01). Vildagliptin treatment reduced the blood glucose levels before and after glucose load, and the area under the curve of blood glucose (P < .01). Serum insulin levels, HOMA-IR, and IL-6 levels in the diabetic rats were higher than that in the normal controls (P < .01), Vildagliptin reduced the serum insulin and IL-6 levels, alleviated insulin resistance, and increased serum GLP-1 in diabetic rats (P < .05).	95
**Thiazolidinediones**								
Denmark 2021	Eight weeks-old male B6.BKS(D)-Leprdb/J (*db/db*) mice.	24	**Intervention**: Rosiglitazone. **Control**: Placebo.	8 weeks	**Primary**: This study was to characterize local gut microbiome and intestinal transcriptome responses in diabetic *db/db* mice following rosiglitazone treatment.	*Lactobacillaceae* and *Lachnospiraceae* were predominant in the small and large intestines, respectively. While *Lactobacillus* was the most relatively abundant genus (>75% of all genera) in the small intestine, the large intestine was characterized by a more diverse genus composition predominantly composed of *Kineothrix, Lactobacillus*, and *Blautia.*	Plasma insulin levels remained stable throughout the entire dosing period in vehicle controls (baseline: 6999 ± 327 pg/mL; termination: 7628 ± 1076 pg/mL, p = .570) and rosiglitazone-treated db/db mice (baseline: 5988 ± 295 pg/mL; termination: 5718 ± 841 pg/mL, p = .741). Rosiglitazone significantly improved glucose excursions in two successive OGTTs performed on treatment day 28 (p < .001) and 49 (p < .001). Rosiglitazone also improved weekly fasting blood glucose levels and terminal HbA1c levels (p < .001).	79
**Sulfonylureas**								
Netherlands 2020	Male and female (postmenopausal). 35–75 years old. BMI scores > 25 kg/m^2^. T2D.	44	**Intervention1**: Dapagliflozin. **Intervention2**: Gliclazide.	12 weeks	**Primary**: This study was to evaluate the effects of 12-week treatment with the SGLT2 inhibitor dapagliflozin and sulfonylurea gliclazide on gut microbiome composition in T2D patients treated with metformin.	Differential abundance analysis yielded no significant shift in specific microbes.	Both dapagliflozin and gliclazide similarly improved glycemic control (p < .001), while dapagliflozin reduced gliclazide slightly increased fasting insulin (p = .011 and p = .569, respectively).	81
**Antibiotics**								
Netherlands 2022	Male with PreT2D and BMI was 31.0 ± 0.5 kg/m2. Male C57BL/6 J mice and 6-week-old male mice.	57	**Intervention**: amoxicillin, vancomycin. **Control**: placebo.	7 days	**Primary**: This study was to explore the effects of the gut microbiota on the function of the exocrine pancreas and the gut endocrine system.	Gut microbiota alters host intestinal proteome. Proteolysis-related proteins, such as elastase and dipeptidase 1, were increased by the HFD, whereas proteins involved in iron homeostasis, such as ferritin heavy chain, were reduced with the HFD. Notably, serine protease inhibitors (serpin) proteins, which have been previously linked to metabolic diseases, were one of the main groups affected by diet and gut microbiota.	In the donor mice, the HFD caused a decrease in circulating GLP-1 levels, and this was reversed with vancomycin treatment. Transferring microbiota from antibiotic-treated mice to GF mice was sufficient to transfer significant changes in GLP-1 secretion.	101
Netherlands 2016	Male with overweight and obese. 35–70-year-old Caucasian.	57	**Intervention**: amoxicillin, vancomycin. **Control**: placebo.	7 days	**Primary**: This study was to investigate how gut microbiota manipulation by antibiotics (7-day administration of amoxicillin, vancomycin, or placebo) affects host metabolism in 57 obese, prediabetic men.	The fecal microbiota composition showed that 7-day vancomycin markedly decreased microbial diversity (p < .001), whereas this was not affected by amoxicillin (p = .42) as compared to placebo. vancomycin decreased the relative abundance of mainly Gram-positive bacteria of the Firmicutes phylum. Among the most strongly affected groups were genus-like groups that contain known butyrate-producing species from *Clostridium clusters IV and XIVa*, such as *Coprococcus eutactus, Faecalibacterium prausnitzii*, and *Anaerostipes caccae*, as well as species involved in BA dehydroxylation such as *Clostridium leptum*. Conversely, Gram-negative *Proteobacteria*, members of *Clostridium cluster IX*, and vancomycin resistant Gram-positive Bacilli such as *Lactobacillus plantarum* and *Enterococcus*, showed increased relative abundance after vancomycin treatment.	Antibiotic treatment did not significantly alter Rd as compared to PLA. Additionally, no effects were found on hepatic and adipose tissue insulin sensitivity, as determined by the insulin-mediated suppression of endogenous glucose production (EGP) and plasma-free fatty acid (FFA) concentrations. In accordance, antibiotic treatment neither altered whole-body insulin sensitivity (HOMA-IR) immediately after cessation of treatment nor at 8 weeks follow-up.	102
**Bariatric surgery**								
France 2022	Male and female T2D with Roux-en-Y gastric bypass.	136	**Intervention**: Roux-en-Y gastric bypass.	5 years	**Primary**: This study was to decipher the participation of the GM in the long-term improvement of T2D after bariatric surgery, as well as its implication in the severity of the persisting cases of T2D.	The more severe cases of unresolved T2D were associated with a major increase in the class Bacteroidia, including 12 species comprising *Phocaeicola dorei, Bacteroides fragilis*, and *Bacteroides caecimuris*. A key observation is that patients who underwent major metabolic improvements do not harbor this enrichment in Bacteroidia, as those who presented mild cases of T2D at all times.	The prevalence of patients in the Severe cluster decreased from 55% at baseline to 30% at 5 years, confirming an overall decrease in T2D severity after RYGB.	109
Portugal 2021	Male and female, age ≥ 20 and ≤ 65 years old; BMI ≥ 30 and < 35 kg/m2; previous diagnosis of T2D.	20	Intervention: Roux-en-Y gastric bypass surgery. Control: Standard medical therapy.	12 months	**Primary**: This study was to evaluate gut microbiota changes after metabolic surgery *versus* standard medical therapy in diabetic adult patients with class 1 obesity.	*Ruminococcus, unclassified_Lachnospiraceae*_ family, and *Faecalibacterium* significantly decreased, while *Klebsiella, Gammaproteobacteria, Enterobacter, unclassified_Gammaproteobacteria, unclassified_Veillonellaceae* increased after 12 months of RYGB. In the medical arm, *unclassified_Lachnospiraceae* and *Sutterella* significantly decreased, while *unclassified_Clostridiales* and *unclassified_Bacteria* increased, when comparing baseline with M12.	The fasting glucose, insulinemia, C-peptide, and HOMA-IR were significantly lower in the surgical arm (*p* = .007, *p* = .020, *p* = .020, and *p* = .027, respectively), and HDL-C was higher (*p* = .004). The surgical arm, 5 participants (62.5%) experienced remission from their diabetes (*p* = .007 for comparison with the medical arm.	108

T2D: Diabetes Mellitus type 2; a-LCD: almond-based low carbohydrate diet; LFD: Low-fat diet; GLP-1: glucagon-like peptide 1; BMI: body mass index; SCFA: short-chain fatty acids; SIT: sprint Interval; MICT: moderate-intensity continuous training; HFD: high-fat diet; STZ: streptozotocin; db/db: diabetic; SGLT2: sodium-glucose co-transporter-2.

### Exercise

Physical activity is an effective strategy to control diabetes; however, its benefits for metabolic homeostasis remain poorly understood.^71^ Recently, a modulating effect of exercise on the GM was reported in humans and animals, both with T2D.^72^ For example, murine models subjected to physical training simultaneously promoted an increase in the abundance of *Bacteroidetes* and a reduction in *Firmicutes* and *Proteobacteria*. Furthermore, individuals with physical activity had greater diversity in microbiota composition and better metabolic capacity than sedentary subjects.^73^

At the physiological level, the effect of physical exercise on GM composition has been associated with reducing inflammatory markers and metabolic endotoxemia, increasing the production of SCFAs, and degrading BCAAs.^74^ Notably, Y. Liu et al. reported that the relative abundance of *Firmicutes, Bacteroidetes*, and *Proteobacteria* was significantly altered after 12 weeks of exercise in a human cohort. In addition, the species belonging to the genus *Bacteroides* (producers of SCFAs) increased significantly, suggesting that physical exercise for short periods of time exerts differential modulating effects on microbial composition.^75^ Moreover, chronic physical activity modifies GM composition and reduces intestinal permeability and systemic inflammation.^76^ On the other hand, Motiani et al. reported the effects of continuous training of different intensities (moderate intensity and speed interval training on intestinal metabolism) on the GM in subjects with T2D and pre-T2D. Remarkably, the authors concluded that the composition of the GM changed due to physical exercise in two weeks. Both forms of training decreased the *Firmicutes/Bacteroidetes* ratio, *Blautia spp*., and *Clostridium spp. Faecalibacterium* increased only in continuous moderate-intensity training. Notably, these changes in microbiome abundances were correlated with clinical parameters. For example, a lower abundance of *Blautia* was associated with better insulin sensitivity. Overall, regular physical activity provides metabolic benefits, even in short periods of time^73,77^ (see Table 1).

### Drugs

Patients with T2D are clinically treated with various anti-diabetic drugs^78^ that normalize blood glucose by targeting different organs and through different mechanisms. For example, GLP-1 analogues stimulate insulin secretion and keep pancreatic β-cells healthy and proliferating. Inhibitors of intestinal hormones, such as dipeptide-4 (DPP-4), suppress appetite in the brain. On the other hand, sodium-dependent glucose transport-2 inhibitors (SGLT2) block renal reabsorption of glucose. Metformin (a biguanide) reduces hepatic gluconeogenesis. Meanwhile, thiazolidinediones (TZD) agonists of PPAR-γ increase glucose uptake in skeletal muscles and adipose tissues.^79^ Last, sulfonylureas (SUs) increase pancreatic insulin secretion.^78,80,81^ The effect between the GM and anti-diabetic drugs is bidirectional; the drug influences microbiota composition, and in turn, the metabolism of the microbiota may have a positive effect in the host.^80^ One effect of these drugs in the host is to modify the gut microbiome composition by increasing the bacteria that produce SCFAs.^82^ In turn, the GM and SCFAs exert effects on anti-diabetic agents, influencing their pharmacogenetics and bioavailability.^80^ Understanding the bidirectional drug-microbiome interaction and how it influences clinical outcomes in T2D patients is necessary to identify possible modulating mechanisms of the GM.^80^ Given their current application, we discuss and present some evidence of the interactions between the GM and three anti-diabetic drugs (biguanides, GLP-1 receptor antagonists, and DPP-4 inhibitors).

Among the biguanides, metformin is used as the first-line treatment in patients with T2D.^83^ Various studies on mouse and human models suggest that metformin increases the abundance of *Akkermansia* and other bacteria that produce SCFAs (*Allobacum, Bacteroides, Blautia, Butyricoccus*, and *Phascolarctobacterium*). Simultaneously, with this dysbiosis, metformin improves glucose concentrations in the patient.^84^ Forslund et al. in their study with T2D patients stratified by treatment regimens, found that the subjects treated with metformin changed their GM composition. They observed that patients treated with metformin presented with a higher production of butyrate and propionate than untreated patients due to the enrichment of bacteria producing SCFAs (*Blautia, Bacteroides, Butyricicoccus, Bifidobacterium, Prevotella, Megasphaera*, and *Butyrivibrio*).^85^ On the other hand, Shin et al. showed statistically significant differences in the abundance of *Firmicutes* and *Bacteroidetes* between mice fed a high-fat diet and treated with and without metformin.^86^ At the genus level, an increase in *Escherichia* and a decrease in *Intestinibacter* were recognized in mice treated with metformin.^86^ In addition, Na-Ri Shin et al. explored the relationship of the anti-diabetic effect of metformin and GM composition in obese mice with T2D. They found that the effect of metformin may be mediated by a specific subset of bacterial taxa associated with an increased abundance of *Anaerotruncus, Lactococcus, Akkermansia, Parabacteroides, Odribacter, Alistipes, Lawsonia, Blautia*, and *Lactonifacter*, of which *Akkermansia w*as responsible for the greatest phylum *Verrucomicrobia* whose abundance was observed in T2D-induced mice treated with metformin compared to control mice with p < .05. Similarly, the authors concluded that the number of goblet cells producing mucin and mucin-degrading bacteria was higher in the group of mice with T2D and treatment with metformin vs the control group (9.5 ± 0.5 vs 6.6 ± 0.3, p > .001). Another outstanding conclusion is that the modification of GM composition was associated with greater glucose tolerance in mice treated with metformin than in control mice (p < .05).^86^ The previous results coincide with those found by de la Cuesta-Zuluaga et al. who studied 14 subjects with a diagnosis of T2D treated with metformin, and 42 healthy individuals with similar clinical conditions.^87^ They concluded that *Akkermansia muciniphila* and *Butyrivibrio* increased their abundance in subjects with T2D respect to the control. In addition, they reported a non significant change in abundance for *Roseburia, Subdoligranulum*, and *Faecalibacterium*, all of which are butyrate-producing bacteria.^87^ Treatment with metformin, in addition to increasing *Akkermansia muciniphila* in the colon, also increases the abundance of *Lactobacillus* in the upper small intestine, which may contribute to the anti-diabetic effect of metformin.^88,89^ Furthermore, metformin increases the population of SCFA-producing bacteria (*Alobacum, Bacteroides, Blautia, Butyricoccus*, and *Phascolarctobacterium*) in the gut.^90^ Taking into account all these findings in animal models and humans, the drug’s ability to modify bacterial diversity becomes evident by selectively increasing the abundance of specific bacteria and altering multiple metabolic pathways in the GM, such as those involved in the metabolism of glucose.

A second frequent drug clinically applied in T2D is the GLP-1 analogue. These activate GLP-1 receptors and increase resistance to inactivation by the DPP-4 enzyme. Currently, six drugs are clinically approved in this category: exenatide, liraglutide, semaglutide, albiglutide, lixisenatide, and dulaglutide.^37^ GLP-1 receptor agonists (liraglutide) can modulate the GM.^91^ Zhang et al. in a murine model, determined the effect of liraglutide (0.4 mg/kg/day) on GM composition. In this study, 58 bacteria changed significantly between the normoglycemic, diabetes-induced, and liraglutide groups (p < .05). Of these microorganisms, 11 showed statistically significant differences between the treatment and control groups. The genera *Flavonifractor, Lachnoclostridium, Ruminococcus_gnavus, Flavonifractor_plautii*, and *Bacteroides_acidi-faciens* were significantly elevated in the liraglutide treated group compared with the diabetic group. The bacteria that significantly reduced their abundance were the genera *Christensenellaceae_R_7_group, Ruminococcaceae_UCG_010, Ruminoclostridium_6, Prevotella_9*, and the class *Mollicutes*. The *Bifidobacterium* and *Lactobacillus* genera were exclusively identified in the liraglutide group compared with the healthy group.^92^ Complementarity, Charpentier et al. evaluated the effect of liraglutide (intervention), exendin (control), and saline solution (control) in a situation of mouse model-induced hyperglycemia. Liraglutide significantly improved insulin secretion, and this effect was associated with changes in GM composition. The frequency of the *Bacteroidetes*/*Firmicutes* phyla relationship increased in response to both GLP-1 receptor agonists. Additionally, *Porphyromonadaceae* and *Lactobacillaceae* significantly increased, while *Lachnospiraceae* and *Bacteroidaceae* decreased in abundance. Exendina modified the families *Lachnospiraceae* and *Porphyromonadaceae*, the genus *Odoribacter*, and *Lactobacillaceae* and liraglutide modified *Escherichia* and *Shigella*.^91^

Finally, dipeptidyl peptidase-4 inhibitor (DPP-4i) is a proteolytic enzyme found in the cell membrane of most cells in the body whose main function is to inactivate GLP-1.^83^ Inhibiting DPP-4 prolongs the circulating half-life of GLP-1, thereby improving its insulinotropic and glucoregulatory capabilities.^93^ Five drugs have so far been approved in this class: sitagliptin, saxagliptin, vildagliptin, linagliptin, and alogliptin.^93^ In particular, the composition of the GM can be modulated with DPP-4 inhibitors.^80^ Xinleng Yan et al. in their study using an animal model, determined the effect of sitagliptin on the composition of GM and its relationship with glucose intolerance. The results obtained are conclusive; treatment with sitagliptin for 12 weeks modified the GM composition, reduced *Firmicutes* (63.19% vs 83.56%, p < .01), and increased *Bacteroidetes* (32.46% vs 16.06%, p < .01). Additionally, serum glucose was reduced in comparison to mice with T2D without pharmacological treatment, showing statistical significance (p < .01).^94^ Also, Zhan et al. determined the effect of vildagliptin on the increase in butyrate-producing bacteria in mice with T2D. Vildagliptin was administered in two doses; high (0.02 g/kg) and low (0.01 g/kg). Statistically significant differences were found in comparison to control rats in the number of operational taxonomic units (OTU), Shannon’s index, and Chao’s index, all with p < .01. Vildagliptin modified the characteristic *Firmicutes*/*Bacteroidetes* index in the GM in mice with T2D, reduced *Firmicutes*, and increased *Bacteroidetes*, butyrate-producing bacteria, and *Lactobacillus*. In addition, vildagliptin treatment enriched the phyla *Streptococcaceae* (p < .01) and *Bacteroides* (p < .01), but decreased *Ruminococcaceae oscillibacter* (p < .05), *Ruminiclostridium* (p < .05), *Anaerotruncus* (p < .01), *Eubacterium* (p < .05), and *Prevotellaceae* (p < .05). It was determined that the animal model treated with vildagliptin at any dose reduced fasting, postprandial glucose, HbA1c, HOMA-IR, and IL-6 compared to T2D and normoglycemic mice, with statistical significance of p < .01 for each variable.^95^

On the other hand, Lin-Wang et al. compared the GM structural modulation of body weight and serum glucose with two treatments: liraglutide (GLP-1 receptor agonist) and saxagliptin (DPP-4 inhibitor). The authors found that mice treated with liraglutide enriched the genera *Allobacum* (p = .004), and *Turicibacter* (p = 1.77e-8), the family *Erysipelotrichaceae*, specifically the genera *Anaerostipes* (p = 5.51e-5) and *Blautia* (p = .039), and the family *Lachnospiraceae* genus *Lactobacillus* (p = .013). These changes in GM composition were associated with a reduction in body weight in mice treated with liraglutide. In contrast, the GM composition in treatment with saxagliptin increased the abundance of some bacteria from the class *Erysipelotrichaceae*, such as *Lactobacillus* (p = .023), *Allobaculum* (p = .017), and *Turicibacter* (p = .001). Furthermore, the abundance of *Bacteoridetes* decreased, specifically the genera *Bacteroides* (p = .003) and *Prevotella* (p = .018). The random glucose concentration was lower in linagliptin-treated mice, p < .05. Liraglutide and saxagliptin were shown to enrich *Lactobacillus* and *Turicibacter*. Furthermore, *Lactobacillus* showed inhibitory activity against DPP-4, through increased incretins and glucose homeostasis.^96^

### Antibiotics

Antibiotics are administered to combat pathogens. However, their use disturbs the microbial composition of some important genera participating in immune, endocrinological, and metabolic functioning.^97^ The use of antibiotics has been associated with remarkable metabolic alterations, mainly when the application comprises broad-spectrum antibiotics and their use occurs in the first years of life. It has been estimated that antibiotics impact the abundance of 30% of the GM, producing a rapid and significant decline in richness, diversity, gene expression, and protein and metabolic activity.^98^ Despite the GM metabolically responding under this perturbation, the initial state is not fully recovered, and antibiotic-induced microbial alterations can remain for months or even years.^99^

Recently, murine model studies and human clinical trials have shown that antibiotics can modulate the GM with antibiotic treatment, which in turn, could reduce glucose intolerance, adiposity, and adipose tissue inflammation. Using a murine model, Fujisaka et al. determined the effect of antibiotics on the GM and host metabolism. In this study, cB6J, 129 T, and 129 J mice were treated with a placebo, vancomycin, or metronidazole in their drinking water. Vancomycin treatment lowered the relative abundance of *Firmicutes* in B6J mice to 37% *(p *= .009) and in 129 T mice to 50% of untreated HFD levels (*p*= .003). This dysbiosis was associated with a rise in the relative abundance of *Proteobacteria*.^100^ In 129 J mice, metronidazole and vancomycin markedly diminished *Verrucomicrobia* from 66 to 0% (p = .002) and 23% (p = .007), respectively, favoring the predominance of *Proteobacteria* and *Firmicutes*. HFD B6J mice treated with vancomycin and metronidazole exhibited reduced blood glucose levels and improved glucose tolerance during the OGTT.^100^ Furthermore, Gridhar et al. evaluated the effect of antibiotic treatment and an HFD on the metabolism and function of the pancreas. The C57BL/6 J mice were divided into four groups: 1) fed a standard chow diet, 2) an HFD for six weeks, 3) fed a standard diet plus oral vancomycin, and 4) metronidazole in the last two weeks of the experiment. Vancomycin treatment significantly increased glucose tolerance and insulin sensitivity in HFD-exposed mice.^101^ On the other hand, Reijnders et al. investigated how GM manipulation by antibiotics (a 7-day administration of amoxicillin, vancomycin, or placebo) affects host metabolism in 57 obese and pre-diabetic men. This study was a double-blind controlled clinical trial in which the population was randomized into three groups: the placebo, amoxicillin, and vancomycin. Vancomycin at seven days decreased the diversity of intestinal microbiota compared to the placebo (p < .001); however, amoxicillin did not affect diversity (p = .42). Likewise, the group treated with vancomycin decreased the relative abundance of butyrate producing bacteria, such as *Coprococcus eutactus, Faecalibacterium prausnitzii*, and *Anaerostipes caccae*, as well as species involved in BA dehydroxylation, like *Clostridium leptum*. In contrast, patients treated with amoxicillin did not experience a change in the composition of the microbiota after seven days of treatment.^102^ The data obtained in this clinical trial were in contrast with several previous studies on rodents, which indicated that antibiotic treatment can improve glucose homeostasis and metabolic alterations.^103^ In human species, the effects of antibiotics on glycemic control or insulin sensitivity remain inconclusive.^102^

### Bariatric surgery

Bariatric surgery (BS) refers to surgical procedures designed to achieve weight loss and long-term glycemic control in patients with T2D and obesity. Importantly, it can achieve better outcomes than non-surgical interventions (medication and diet).^104^ The two most common procedures in BS are sleeve gastrectomy (SG) and Roux-en-Y gastric bypass (RYGB), both with comparable efficacy. According to the American Diabetes Association (ADA) and the International Diabetes Federation (IDF) guidelines, BS is recommended in individuals with T2D and body max index (BMI) of at least 35 kg/m2, it can be an option for individuals with mild obesity (BMI of 30–34.9 kg/m2) who have inadequate glycemic control despite optimal medical management.^104^

Noticeably, BS can achieve a remission of T2D in 23 to 60% of patients, defined by normalization of blood glucose (FPG below 126 mg/dL or estimated HbA1c below 6.5%) without the need for normoglycemic medications.^104^ After BS, an improvement in metabolism occurs even before weight loss begins. Although the mechanisms underlying these favorable responses are not fully understood, the gut neuroendocrine system, gut hormones, bile acids, and GM have been proposed as key mediators.^105^

Remarkable changes in GM composition were observed after BS. In general, patients treated with BS experience an early increase in the richness of the GM, which may reflect an attempt to restore intestinal homeostasis. There is also a profound shift in certain genera associated with an improvement in glucose metabolism and a reduction in systemic inflammation markers. A comparative study of GM composition in T2D patients before and after BS showed an increase in the relative abundance of *Escherichia, Klebsiella*, and *Akkermansia muciniphila* and a decline in the relative abundance of *Faecalibacterium prausnitzii, Lactobacillus acidophilus*, and *Coprococcus*.^106^ Different BS procedures affect GM differently, with the most notable change occurring in RYGB. Sanchez-Alcoholado et al. found that patients after SG experienced an increase in the relative abundance of *Akkermansia, Eubacterium, Haemophilus and Blautia*. In contrast, patients after RYGB saw a preferential increase in the relative abundance of *Veillonella and Granucatiella*.^107^ In addition, the microbiota are altered in individuals with mild obesity treated with RYGB, with a rise in the relative abundance of *Klebsiella, Gammaproteo-bacteria, Enterobacter, Gammaproteobacteria_un-classified, and Veillonellaceae_unclassified*.^108^

The role of the GM in the prognosis of T2D remission after BS has also been studied. J. Debédat et al. demonstrated that an inadequate response to RYGB in T2D patients is associated with an increase in the *Bacteroida* class (such as *Bacteroides fragilis species, Bacteroides vulgatus, Phocaicola dorei, and Bacteroides caecimuris*) before and five years after surgery compared to post-RYGB patients with adequate remission. In addition, they investigated the causal link between GM and BS by performing human-to-mouse (free mice) FMT. They found that the phenotype of IR can be induced in a recipient mouse by receiving a transplantation of fecal microbiota from patients with inadequate remission after RYGB.^109^

Several microbiota-mediated mechanisms have been proposed to induce glycemic control and improve insulin sensitivity after BS. One of them is the crosstalk between the BAs and the GM. After BS, there is an increase in the blood concentrations of primary BAs and secondary BAs in T2D patients. Altogether, the overproduction of these BAs impacts lipid and glucose metabolism, and seems to be associated with the remission and improvements of T2D patients treated with BS.^110^ However, these outcomes are in contrast to those reported by Ilhan et al. who characterized the fecal metabolome of an American cohort with severe obesity and T2D after BS (the majority of subjects had resolution of diabetes and other comorbidities 12 months after treatment). They found a decline in the concentration of secondary BAs at 12 months after surgery compared to the non-surgical controls.^111^ Overall, these results imply that GM and BS have an important codependence in the improvement of T2D patients. Consequently, the study on the differences in GM composition is critical for understanding the pathways underlying metabolic improvement after surgery.

### Fecal microbiota transplantation

FMT has become an outstanding research topic with potential applications in clinical medicine and biomedicine.^112^ Recently, FMT has been proven to be an effective method for treating and preventing the recurrence of gastrointestinal disorders through host-microbiota interactions; for example, FMT treatment against *Clostridium difficile* infection.^113^ Furthermore, FMT has been suggested as a therapeutic approach to modulate chronic and metabolic conditions such as T2D.^114^ Vrieze et al. established the effects of infusing GM from lean donors to male recipients with metabolic syndrome on the recipients’ microbiota composition and glucose metabolism. Six weeks after microbiota infusion, receptor insulin sensitivity increased along with butyrate-producing bacteria levels.^115^ Likewise, the authors demonstrated that FMT with or without lifestyle changes increased butyrate-producing bacteria in subjects with obesity and T2D. A recent study highlighted that the combination of lifestyle changes and FMT increased *Bifidobacterium* and *Lactobacillus* compared to FMT alone.^116^ In addition, Kootte et al. studied the effect of allogeneic FMT (from lean donors) on metabolism in relation to GM composition at 6 and 18 weeks after treatment. Moreover, the authors obtained microbiome composition in autologous FMT as the control treatment (placebo) in the donor subjects. In their clinical trial, the authors observed a statistically significant increase in insulin sensitivity after 6 weeks of allogeneic transplantation, accompanied by an altered composition of the GM. Further, using the GM composition, it was possible to classify the status of responders versus non-responders to the allogeneic FMT (recipient operating characteristics [ROC] AUC 0.88). Metabolic responders to FMT were characterized by lower baseline GM diversity and a higher abundance of *Subdoligranulum variabile* and *Dorea longicatena* than non-responders, while the abundance of *Eubacterium ventriosum* and *Ruminococcus torques* was lower in the baseline fecal samples. Although FMT has been proposed as a candidate to treat T2D, the molecular mechanisms underlying the therapeutic benefits are not yet understood.^116^

On the other hand, in a murine model with T2D, FMT increased the number of species and the alpha diversity indices (Shannon and Simpson). In addition, the level of HbA1c decreased and improved pancreatic β-cell function (measurement performed with HOMA-B) in the FMT group. Notably, this study suggests that GM transplantation reverses IR and damages islets.^117^ Additionally, Zhang et al. determined the effect of transplanted fecal bacteria from Kazakhs (the Kazakh Chinese ethnic group) with normal glucose tolerance on male db/db mice with T2D. In these recipient mice, the levels of *Desulfovibrio* and *Clostridium coccoides* in the intestine were significantly reduced, but the abundance of *Akkermansia muciniphila* and the expression of colonic protein histone deacetylase-3 (HDAC3) in the colon samples were increased. These results indicate that *Akkermansia muciniphila* could have affected the metabolism of the murine model by regulating the expression of HDAC3. This is because it can activate brown fat cells to oxidize lipids, increase metabolism and promote weight loss to counter T2D. Furthermore, in this study, HDAC3 was positively correlated with glycolipid levels, suggesting that *Akkermansia muciniphila* may be the main intestinal probiotic that improves metabolism in T2D.^118^ Based on previous evidence, FMT has been recognized as a therapeutic strategy that in combination with lifestyle can be potentially effective in the treatment of T2D.^119^ However, more evidence in humans needs to be generated, and we suggest that for future interventions, determining the baseline fecal microbiota composition may help predict treatment efficacy.^112^

### Phageome

Obesity and T2D are associated with changes in gut bacterial composition, but little is known about the role of the virome in T2D disease development. Yang et al. reported the results of viral-bacterial transkingdom correlation for a Chinese cohort of 101 lean controls and 128 obese subjects (74 diagnosed with T2D). The authors found a decreased number of correlations between the relative abundance of the virome and bacteriome in obese subjects compared to the lean controls. Furthermore, obese subjects with T2D displayed an increased number of negative correlations and a lower number of positive correlations compared to the lean controls.^120^

Given the extensive evidence that phages can shape the composition and function of bacterial communities, the phageome of the human gut has been studied in T2D patients. For example, Yingfei et al. conducted a computational study of associations (with SparCC, a tool to infer correlation networks) between bacterial and phage abundance in a Chinese cohort of 74 healthy patients and 71 T2D patients.^121^ Their results imply that the number of phages in the intestinal tract of diabetes subjects was significantly increased, especially in the group with seven phage OTUs (pOTUs) (*Siphoviridae* phage family for *Lactobacillus, Listeria* and *Staphylococcus*). However, they identified pOTUs belonging to the *Caudovirales* order, which has several limitations of taxonomic annotation. Despite these incipient achievements providing little insight into the mechanisms by which the phageome participates in T2D, they supply evidence of their involvement in the disease and pave the way for further research to discover human gut phage functions in the development of T2D.^121^

## Systems biology: In silico modeling of metabolism in gut microbial communities

**Figure 4.**
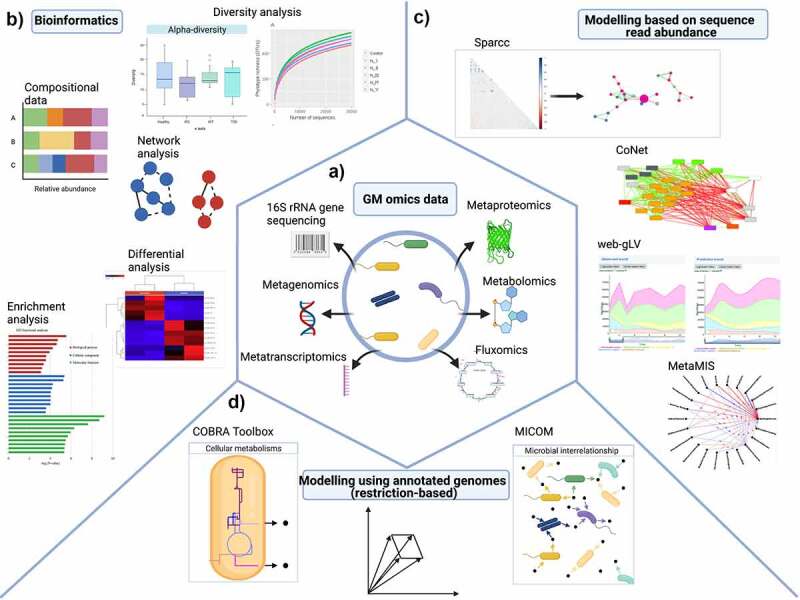
SB approaches used with omics datasets from T2D. a) Data collection by HT technologies, b) Bioinformatics, c) Modeling based on sequence read abundance, and d) Modeling by using annotated genomes (restriction-based). Adapted from^116–121^ Created with BioRender.com.

High-throughput technologies (HTs) are powerful tools, as they offer new insight into the functioning and behavior of microbial communities. However, data is not sufficient, and the generation of knowledge requires computational schemes with the capacity to integrate data and reach conclusions at a systemic level. As depicted in Figure 4, data obtained from HT can be used as input in several systems biology (SB) tools to provide insight into the structure and function of microbial communities.^122^ For example, proteomic studies and 16S rRNA data suggest a lower abundance of the *Lachnospiraceae* family in obese diabetic mice than in the control group.^123^ Additionally, Reeves et al. suggested that inoculation with *Lachnospiraceae* in germ-free mice is associated with suppression of *Clostridium difficile* colonization.^124^ Altogether, these findings suggest that an increase in the abundance of *Lachnospiraceae* can be considered beneficial and is highly associated with the maintenance of gut homeostasis and health.^123^ On the other hand, integration of whole metagenome shotgun (WMS) with metabolomics pointed out that a high fiber diet was associated with a decrease in the abundance of sulfate reducer bacteria and a rise in SCFA producers, which in turn is related to better levels of HbA1c and increased GLP-1 in the host.^125^ Moreover, an integrative analysis with WMS and targeted metabolomics performed on a T2D-Chinese cohort supplies evidence that SCFA metabolism enriches its activity after metformin application. Simultaneously, this last finding was associated with higher levels of butyrate and propionate metabolites.^88^

In terms of the development and progression of patients with T2D, the information obtained through HT is in constant growth. For example, Zhou et al. carried out deep profiling of the transcriptomes, metabolomes, cytokines, proteome, and GM of a cohort of 106 subjects (healthy and pre-T2D). This report revealed two main conclusions about GM and individuals with prediabetes. First, GM is differ between healthy and IR subjects. Second, the GM (conceived as a holobiont) in subjects with IR responded differently than those associated with healthy subjects when the immune system was stimulated (immunization and viral respiratory infections). Moreover, the global co-association analysis revealed precise host-microbe interactions that differed between healthy and IR subjects. In this way, the correlations between GM and host circulating cytokines revealed several interactions in IR subjects (p < .05). For example, IL-1β was positively correlated with *Barnesiella*, and TNF-α was inversely correlated with Faecalibacterium.^126^ Given that the host’s IL-1 activity^127^ and IFN-γ^128^ signaling are related to *Barnesiella* and *Faecalibacterium*, they are associated with TNF-α, which might enhance IL-17 activity.^124^ The absence of these correlations in IR subjects suggests that IR could affect associations between GM and cytokines.^126^ Moreover, the genera *Clostridium-XIVb* and *Phascolarctobacterium* were significantly correlated (p < .05) with *Holdemania* in healthy participants, and *Holdemania* was correlated with *Clostridium-XlVa, Clostridium-XVII* and *Collinsella*. Thus, IR and healthy associations can indicate different patterns of GM interactions between the two groups.^126^ These findings support the idea that systemic analyses offer novel insight into the role of GM in the development and course of T2D disease.

An effort to discover new insights about the function, structure, and design of GM has been triggered by the development of qualitative and quantitative computational models.^129^ Due to their relevance to elucidate regulatory and metabolic mechanisms, we discuss the main computational approaches that have been used to explore the relationship between GM and T2D, which are summarized in Figure 4.

### Bioinformatic and functional studies

The generation of extended comprehensive studies for GM requires massive high-quality data and bioinformatic methods to derive a proper biological interpretation. For example, one of the first and most fundamental questions belongs to the taxonomic composition of the GM and the phylogenetic connections of its members. An approach for taxonomic classification is based on the counts of specific marker genes., i.e., 16S rRNA, 18S rRNA, and internal transcribed spacer. These approaches have enabled the analysis of different and complex habitats in the GM. In particular, 16S rRNA has been frequently used due to its capacity to analyze large numbers of samples, i.e., multiple patients and longitudinal studies.^130^

At the same time, several bioinformatics tools have been developed to examine the reads obtained from 16S rRNA technologies, most of which rely on three main steps: preprocessing and quality control, taxonomic assignment, and ecological analyses.^131^ After the primary analysis, it is possible to perform more specific analyses, (such as those focused on enrichment, differential abundance, and association networks), at several taxonomic levels (e.g., genus, family).^132^

The taxonomic assignment is a key step in the 16S rRNA sequencing data analysis pipeline. Currently, three representative tools have been successfully and widely applied in 16S analysis: Quantitative Insights Into Microbial Ecology (QIIME2), Mothur, and DADA2. In general, these tools have reliable and comparable results in terms of performance for 16S analysis under different next generation sequencing technologies.^133^

Although 16S analysis allows us to obtain information on the taxonomic and phylogenetic community structure at the genus level, these methods cannot provide information about the functional genes in the bacterial community. Despite this limitation, there are bioinformatic strategies to fill this gap. Phylogeny is actively correlated with functional gene patterns;^134^ thus, we can predict functional genetic information through diverse bioinformatics tools, such as the phylogenetic investigation of communities by reconstructing unobserved states (PICRUSt2) and Tax4Fun.^135^

However, we need to consider that HT data (e.g., 16S rRNA reads) fall into a class of data termed “closed” or “compositional”, which includes particular geometric and statistical properties; this makes establishing microbial taxa associations between communities challenging.^136^ Hence, the following bioinformatic tools have been developed for statistical analysis of microbiome data, such as LEfSe and MaAsLin2.^137^ They provide several methods for data normalization and transformation. Additionally, SparCC, and SPEIC-EASI address the compositional problem by assuming that few species are correlated, and BAnOCC makes no assumptions about the microbial data.^138^ In addition, several supervised learning algorithms have been proposed to identify a subset of highly predictive taxa from the different stages of the disease. Due to the high complexity of the data, it is necessary that these algorithms be able to model the complex interactions and non-linear effects between microbial communities. As such, the most commonly used methods are support vector machine, random forest, and multilayer perceptron with variable predictive accuracy.^139^

### Modeling based on sequence read abundance: Inferencing of microbial ecological rules

Among the computational approaches available to analyze the microbiome, we highlight those associated with reconstructing the ecological structure from HT, such as 16S rRNA and WMS, SparCC,^140^ MENA,^141^ LSA,^141^ CoNet,^142^ SPIEC-EASI,^140^ MetaMIS,^143^ and Web-gLV,^144^ among others fall in this category. These bioinformatic tools integrate correlation networks and include methods for OTU pre-processing and microbial associations. The most common output from all of these tools is the inference of a microbial association network and the estimation of its robustness through their topological parameters, such as cluster coefficient, connectivity, and modularity.^145^

By following these strategies, some remarkable studies about T2D and microbiome association have been recently reported (Table 2). For instance, Ross et al. explored the association between GM composition and T2D in a cohort of 63 Mexican-American subjects from Cameron County (CCHC) (Texas, US).^146^ This geographic location was selected due to the high rate of obesity and T2D cases that emerge there each year.^147^ According to this report, the authors identified more reads in *Lachnospiraceae* and *Roseburia* (*Firmicutes*) in CCHC subjects that in the Human Microbiome Project (HMP). Moreover, the authors deduced that *Lachnospiraceae* plays a key role in metabolic functions in the progression of T2D in CCHC subjects.^146^

**Table 2.** T2D analysis reported by modeling based on sequence read abundance approach.

SB/Bioinformatic Tool	Seq- Tech	Population	Associated genera with T2D	Reference
SparCC	Roche pyroseq 454 (V1-V3) 16S	63 patients with T2D and obesity	*Lachnospiraceae*	^146^
SparCC	Illumina MiSeq (V3-V4) 16S 300 bp	450 patients with T2D and hyperlipidemia	*Blautia and Faecalibacterium spp.*	^148^
SparCC	Illumina MiSeq WMS	71 patients with T2D	Siphoviridae**	^121^
SparCC	Illumina MiSeq (V1-V3) 16S 300 bp	106 patients with preT2D	*Barnesiella and* *Faecalibacterium*	^126^
DESeq2	Illumina MiSeq (V4) 16S 250 bp	427 patients with IFG, IGT, IFG+IGT, T2D and NG*	*Escherichia, Veillonella, Blautia and Anaerostipes*	^170^
CoNet	Illumina HiSeq (V3-V4) 16S 250 bp	83 patients with T2D and diabetic retinopathy	*Anaerobiospirillum, Gardnerella, Cloacibacillus and Leptotrichia*	^154^
SparCC	Illumina NovaSeq 6000 WMS and VLP	74 patients with T2D	*Escherichia phage, Geobacillus phage,and Lactobacillus phage*	^120^

*Normal Glucose Tolerance (NG), **A virus family.

In another study using SparCC, it was found that alterations in GM are involved in the treatment of T2D with hyperlipidemia in a Chinese cohort of 450 subjects exposed to two clinical interventions: metformin and AMC (a Chinese herbal formula of *Rhizoma Anemarrhenae, Momordica charantia, Coptis chinensis, Aloe vera*, and red yeast rice).^148,149^ In the metformin-treated group, the authors noted a remarkable increase in *Blautia* spp, (SCFA producer), which is in line with other studies conducted on animals.^90^ Also, they observed a decline in *Akkermansia* in T2D Chinese subjects with metformin treatment. However, these findings do not agree with those of other studies on humans, where *Akkermansia* increases after metformin is consumed.^84,118,150,151^ This discrepancy might be explained when we take into account strain-specific functions; thus, the authors suggested further studies (such as molecular analysis the full ribosomal 16S gene and phylogenetic trees) to clarify this controversy.^148,149^ On the other hand, treatment with AMC increased the abundance of two genera related to butyrate production (i.e., *Faecalibacterium* and *Roseburia*).^152^ Interestingly, *Faecalibacterium prausnitzii has* been reported as a functionally important bacterium to prevent physiological damage through the production of butyrate and anti-inflammatory metabolites.^153^ Moreover, AMC had a stronger modulatory effect on the GM than metformin treatment in terms of improving IR and triglyceride levels. This can be explained by the synergistic effects of the multiple phytochemicals present in AMC.^148^ Concerning other diseases caused by T2D, the information is limited.^154^ Das et al. published an interesting study on the association between GM dysbiosis in T2D and diabetic retinopathy (DR) with an Indian cohort of 30 subjects. Regarding T2D subjects, the authors found positive associations between a chronic low-grade inflammatory state and pathogenic genera such as *Gardnerella, Atopobium, Fusobacterium, Gemella, Halomonas, and Vagococcus*. Moreover, and with DR participants, a reduction in the abundance of anti-inflammatory and probiotic bacteria in comparison to other genera was observed. However, in terms of the GM of T2D and DR, the authors did not report significant differences at the genus level.^154^

Last, related to modeling based on sequence read abundance, generalized Lotka-Volterra modeling enhances the classical predator-prey models of the two species, which are broadly used in ecology.^144^ The main advantage of these models is their capacity to estimate the native growth and interaction constraints of uncultured microbes in a given environment from temporal data.^155^ Once the parameters are delimited, we can analyze the variations in GM composition over time with unidentified initial conditions.

### Modeling using annotated genomes (constrained-based)

Genome-scale metabolic reconstruction and constraint-based modeling is a paradigm in systems biology to quantitatively explore the metabolic activity of a bacterium or a group of bacteria constrained in a specific metabolic environment. This approach is characterized by three global elements: the use of genome-scale metabolic reconstruction for microorganisms, their mathematical representation to simulate their metabolic phenotypes, and their integrative description with HT technologies.^156^ A variety of computational tools based on genome-scale metabolic models (GEMS) has been developed in recent years with the purpose of quantitatively examining metabolic activity in the microbiome. Among these methods, we highlight the COBRA Toolbox and cobrapy, computational frameworks developed to model genome-scale metabolic reconstructions through constraint-based modeling.^157^ Based on this approach, there have been some efforts to characterize the microbial metabolic community at small, medium, and large scales. Among the efforts to model metabolic activity and species-level interactions in GM, we list computational strategies such as OptCom,^158^ cFBA,^159^ CASINO,^160^ BacArena,^161^ Dynamic OptCom,^162^ bialSim,^163^ and MICOM.^9^ These schemes differ from each other in several ways: 1) the amount of genome-scale metabolic reconstruction capable of being included in the simulation, 2) the dynamic condition constraining the state of the system (steady or unsteady state), and 3) the computational procedure to define and calculate the community objective function in the microbial community.^164^

In terms of T2D and metabolic modeling of microbiota through annotated genomes, the information is scarce but continues to growth with several articles.^165^ For example, Rosario et al. used the COBRA Toolbox to model the contribution of four bacteria (*Escherichia spp, Akkermansia muciniphila, Subdoligranulum variabile, and Intestinibacter bartletti*) to the physiology of T2D patients undergoing metformin treatment.^166^ To characterize the metabolic alterations produced by this dysbiosis, they applied flux balance analysis (FBA) coupled with synthetic lethality analysis interactions to identify patterns of growth. Their results suggest that the metabolism of *Escherichia sp., A. muciniphila, S. variabile*, and *I. bartlettii* may explain the features observed in T2D-metformin patients, which are related to commensal and competing behavior through extracellular compounds, including SCFAs, H_2_, and amino acids.

From the other side, Diener et al. (2020) reported MICOM,^9^ an OptCom based framework capable of simulating the GM metabolism of almost 850 instances of genome-scale metabolic reconstruction simultaneously, starting from the relative abundance of bacteria obtained from 16S or metagenome technologies. Remarkably, the authors were able to derive the growth rates that correspond directly to the observed replication rates. Moreover, they integrated several constraints, such as taxon abundance and adjustable dietary input, to prepare personalized metabolic models for individual GM samples. With T2D, T1D, and the healthy control data of 186 subjects, the authors showed that the community-level production of SCFAs was heterogeneous and mostly distinctive at the individual level. Also, their model output showed complex cross-feeding associations among bacteria, which mirrors the complex community structure that is difficult to measure *in vivo.^9^* In addition, MICOM was able to predict reduced SCFA (butyrate and propionate) production levels in T2D participants, with a consecutive restoration of these rates found in subjects with metformin treatment. In general, they reported that changes in taxon abundance or diet have highly personalized effects.^9^ Based on these findings, the in silico modeling of the metabolic activity of microbiota has started to be a fundamental and useful approach to obtain quantitative mechanistic explanations that complement association studies. This last point involves genome-scale metabolic reconstruction of bacterial communities in the gut microbiome and diet; all of these are placed in the context of a personalized background.

## Discussion and perspectives

The prevalence of T2D has become a serious public health problem worldwide.^167^ Genetic components, a sedentary lifestyle, and dietary habits (low dietary fiber and high fat consumption) are etiological factors that contribute to the development of T2D.^168^ Recently, GM dysbiosis has been integrated as a factor associated with the rapid progression from IR to T2D. Notably, this dysbiosis can remodel functions of the intestinal barrier and metabolic pathways in the host. In particular, these alterations are closely associated with SCFA production, bile acid transformation, adipose tissue inflammation, and chronic low-grade systemic inflammation.^169^ In this review, we have discussed in detail how the physiological variables in T2D are associated with a disruption of microbiota in the host, as well as how lifestyle, drugs, and new promising interventions like FMT can control and reshape their profiles to benefit the health of the host. Altogether, these findings offer innovative possibilities for preventing and treating T2D. However, their implementation still faces some challenges that need to be addressed in the coming years. Let us review some of them.

First, inter-individuality variability is an inherent factor that underlines the need to define microbiome profiles at the ethnic, geographic, and sociocultural levels. While in Chinese treatment-naive individuals with T2D *Akkermansia muciniphila, Faecalibacterium prausnitzii* and *Roseburia intestinalis* are relevant to the phenotype,^151^ *Escherichia-Shigella, Veillonella, Blautia* and *Anaerostipes* were associated with T2D progression in a Mexican cohort.^170^ These and other reports provide evidence of the need to characterize the local microbiome, and reinforce the need to generate an individualized intervention to enhance glycemic control through the synergistic effects of lifestyle, diet, probiotics, drugs, or surgical interventions.

Second, dietary components orchestrate and modulate GM composition and thus alter the host’s metabolism.^171^ However, the response to diet differs among individuals, with some being non-responders to dietary interventions,^172^ since what can be a good diet for one person might not work for someone else. Personalized nutrition requires frequent evaluation of anthropometric, biochemical, and clinical variables for designing optimal interventions that produce a favorable change in the patient’s metabolism. Although the idea is clear, the practical implementation of this dietary plan in conjunction with other adjuvant factors, such as drugs and changes in lifestyle, remains a challenge in many countries. Some questions with inconclusive answers should be addressed in the future. For instance, the long-term effect of diet on the patient, and how to design it in terms of socio-cultural factors. Although some contributions have been reported in this aspect,^173^ we consider the implementation of these strategies to represent the vanguard of personalized nutrition.

Third, antidiabetic treatments positively influence the host through changes in GM and the host’s metabolism. For example, metformin can facilitate a healthy state by increasing the abundance of the *Lactobacillus* and *Akkermansia muciniphila* strains.^87^ Other drugs, such as DPP-4 inhibitors, decrease the abundance of *Oscillibacter* and increase the abundance of *Lactobacillus*. These compositional changes reduce TLR ligands and pro-inflammatory cytokines and increase propionate. Although metformin is the first-line treatment in most T2D patients, there is a spectrum of drugs that are administered according to clinical variables defining the individual (such as comorbidities, patient preferences, tolerability, and cost). One challenge here is to define the role of the microbiome in selecting the treatment, which is unclear, because there is a specific pattern of microbiome phenotypes that can increase the success of the treatment. To this end, longitudinal studies that integrate the different omics are necessary to verify the effect on the long-term composition and help clarify the role of microbial ecology in the host. Remarkably, this information can generate valuable information to help decide on precision medical treatments that slow down the progression and long-term vascular complications of the disease, and which increase quality of life for patients with T2D.

Fourth, FMT could be used as a favorable modulator of insulin resistance, showing potential in the treatment of T2D patients. However, the Food and Drug Administration (FDA) has issued several warnings on FMT regarding the potential risk of serious or life-threatening infections. In June 2019, approximately two immunocompromised adults developed invasive infections caused by ESBL-producing *Escherichia coli*, and consequently one died.^174^ Additionally, in March 2020, the FDA reported that two patients developed enteropathogenic *Escherichia coli* (EPEC) and Shiga toxin-producing *Escherichia coli* (STEC) infection after receiving FMT.^175^ Therefore, FMT as an experimental therapy requires a well-defined and standardized methodology to design properly conducted clinical trials in the next few years. Despite their practical implications, information on the mechanisms underlying its modulatory effect on microbiota is currently scarce.

Fifth, beyond HT technologies, SB offers new approaches to mechanistically understand T2D development, physiopathology, and its relationship with GM through the constant development of integrative computational approaches. For example, in the framework of modeling based on sequence read abundance, SparCC (a tool to infer correlation networks) has proven to be an outstanding procedure for estimating correlations in microbiota composition, and it has become the gold standard for association studies related to GM.

On the other hand, constrained-based modeling allows for the versatility needed to simulate bacterial communities in numerous conditions that cannot be performed *in vivo*, such as *Clostridium difficile* infection. In this way, computational modeling of metabolism in microbiota, through software such as MICOM, offers a computational framework to infer the growth rates of selected bacteria and the metabolic interactions into GM. Moreover, it provides a high-throughput platform for generating mechanistic hypotheses and testing them in clinical assays. We suggest that the most useful application of metabolic models in bacterial communities is to offer detailed functional metabolic inferences that can serve as a means for novel hypothesis testing.^170^ Despite these computational themes, which have been applied in GM, their use is pioneering in exploring the metabolic consequences of T2D. The outcomes of such approaches comprise the foundations for developing more accurate models by including information about the GM and their ecological relationships with the host (as a holobiont). For example, Thiele et al. (2020) investigated a complete sex-specific model of humans that involved 26 organs and six cell types in the blood to study inter-organ metabolic fluxes and to explore host-microbe cometabolism.^176^ The accuracy of the hypothesis, generated with genome-scale metabolic reconstruction, the reproducibility, and reuse should be a challenge to face in the future of T2D.

Finally, in recent years, there has been a real revolution in the field of big data. In this sense, real-world data and real-world evidence play a more relevant role in medical care decisions.^177^ A vast amount of health-related data is now being collected and stored due to the increased use of computers, mobile devices, wearable devices, and other biosensors.^178^ These data have the potential to allow us to better design and conduct clinical trials and studies in health care to answer questions that were previously unfeasible. In addition, with the development of new and sophisticated analytical capabilities, we can better scrutinize data and apply the results of the analysis to the development and approval of medical products. For example, in the study of GM, it is necessary to design studies with a sufficient number of subjects of both sexes. In addition, information on lifestyle, diet, mental health, comorbidities, and metadata should be included that allow for comprehensive interpretation of the GM. To this end, it is necessary to have electronic databases at different levels (state, national, and world) for multicenter data analysis. Likewise, the capacity of omics sciences should be increased with shotgun studies, meta-transcriptomics, meta-metabolomics, meta-proteomics, and medium-term fluxomic studies to grasp the dynamics of metabolism in the microbial community and the host.^122^ In conclusion, we now have more measurable variables and are able to add more “data layers” to systems biology studies. Undoubtedly, we are only at the tip of the iceberg of understanding host-microbiome interactions and their specific mechanisms of modulation, one of the frontiers in the medicine of this century.

## Supplementary Material

Supplemental Material
