# Supplementary material for: Type 2 diabetes, gut microbiome, and systems biology: A novel perspective for a new era
Source: Gut Microbes. 2022 Aug 24;14(1):2111952. doi: 10.1080/19490976.2022.2111952 (PMC9423831; doi:10.1080/19490976.2022.2111952)
Supplement: Supplemental Material [file KGMI_A_2111952_SM5588.docx]

**Supporting Information**

**Type 2 diabetes, gut microbiome, and systems biology: A novel perspective for a new era​**

**Martínez-López Yoscelina Estrella^1,2,3^, Esquivel-Hernández Diego A.^1^, Sánchez-Castañeda Jean Paul^1,4^, Neri-Rosario Daniel^1,4^, Guardado-Mendoza Rodolfo^3,5*^, Resendis-Antonio Osbaldo^1,6^*.**

^1^Human Systems Biology Laboratory. Instituto Nacional de Medicina Genómica (INMEGEN). México City, México.

^2^Programa de Doctorado en Ciencias Médicas, odontológicas y de la Salud, Universidad Nacional Autónoma de México (UNAM). Ciudad de México, México.

^3^Metabolic Research Laboratory, Department of Medicine and Nutrition. University of Guanajuato. León, Guanajuato, México.

^4^Programa de Maestría en Ciencias Bioquímicas, Universidad Nacional Autónoma de México (UNAM). Ciudad de México, México.

^5^Research Department, Hospital Regional de Alta Especialidad del Bajío. León, Guanajuato, México.

^6^Coordinación de la Investigación Científica – Red de Apoyo a la Investigación, Universidad Nacional Autónoma de México (UNAM). Ciudad de México, México.

*Correspondence: Osbaldo Resendis – Antonio [oresendis@inmegen.gob.mx](mailto:oresendis@inmegen.gob.mx), Rodolfo Guardado – Mendoza guardamen@gmail.com.

**Table S1.** Descriptive characteristics and assessment of nutritional/dietary interventions in gut microbiota of T2D patients.

| **Nutritional/dietary interventions** | | | | | | | | |
| --- | --- | --- | --- | --- | --- | --- | --- | --- |
| **Country**  **year** | **Participants** | **Sample size** | **Intervention implemented** | **Treatment duration** | **Aims/Outcomes** | **Gut-microbiota significant differences** | **Clinical effect** | **Refer** |
| Japan  2021 | Male and female.  ≥ 30 – 80 years old.  BMI ≥ 25 kg/m^2^.  T2D (treatment with only diet and exercise or drugs). | 88 | **Intervention:** Synbiotic intervention.  **Control:** They did not take any synbiotic. | 24 weeks | **Primary:** This study investigates the effects of synbiotic supplementation on chronic inflammation and the gut microbiota in obese patients with type 2 diabetes.  **Secondary:** This study was to determine the effect of synbiotic supplementation in the gut microbiota. | Relative to the control group, the synbiotic group demonstrated significant positive changes in the changes of *Bifidobacterium adolescentis* and *Bifidobacterium pseudocatenulatum* from baseline to both 12 and 24 weeks, as well as of *Veillonella ratti* from baseline to 12 weeks and of *Bacteroides coprocola* and *Megasphaera elsdenii* from baseline to 24 weeks. | The two groups demonstrated no significant changes in IL-6, LBP, or hs-CRP from baseline to 24 weeks. Regarding glycemic control, the synbiotic group showed significantly higher levels of fasting blood glucose and HbA1c at 12 weeks compared with the control group, and also a significant positive change in HbA1c from baseline to 12 weeks. the change in IL-6 level from baseline to 24 weeks, did not differ significantly between the two groups in the per protocol set analysis. | 1 |
| Germany  2019 | Male and female.  40 – 60 years old.  BMI 28 – 35 kg/m^2^.  T2D treated (lifestyle modification, metformin, or other oral glucose-lowering medication). | 12 | **Intervention:** Isocaloric dietary intervention + AA substitution BCAA+/BCAA-.  **Control:** Isocaloric dietary intervention + AA substitution BCAA-/BCAA+. | 5 weeks | **Primary:** This study was to determine the Lower intake of BCAAs improves tissue-specific insulin sensitivity.  **Secondary:** This study was to determine a lower intake of BCAAs changes the gut microbiota composition. | 11% lower abundance of *Firmicutes* after BCAA^−^ dietary intervention compared with BCAA^+^, whereas the abundance of *Bacteroidetes* was 40% higher (both *P* < 0.05). | After the BCAA− diet, BCAAs were reduced by 17% during fasting (*P* < 0.001), by 13% during HEC (*P* < 0.01), and by 62% during the MMT (*P* < 0.001). Under clamp conditions, whole-body and hepatic insulin sensitivity did not differ between diets. After the BCAA− diet, however, the oral glucose sensitivity index was 24% (*P* < 0.01), whereas meal-derived insulin secretion was 28% lower (*P* < 0.05). | 2 |
| China  2018 | Male and female.  35 – 70 years old.  T2D. | 43 | **Intervention:**  A high-fiber diet is composed of whole grains, traditional Chinese medicinal foods, and prebiotics (W group).  **Control:** Patient education and dietary recommendations based on the 2013 Chinese Diabetes Society guidelines for T2D (U group). | 28 days | **Primary:** This study evaluates the effect of a high-fiber diet on the growth of SCFA-producing organisms in diabetic humans. | The high-fiber diet-induced changes in the entire gut microbe community and correlated with elevated levels of GLP-1, a decline in acetylated hemoglobin levels, and improved blood-glucose regulation. | The level of hemoglobin A1c (HbA1c), decreased significantly  from baseline in a time-dependent manner in both groups; from day 28 onward, however, there was a greater reduction in the W group. The proportion of participants who achieved adequate glycemic control (HbA1c < 7%) at the end of the intervention was also significantly higher in the W group (89% versus 50% in the U group).  There was a temporal difference in fasting blood glucose levels only the W group achieved a significant reduction by day 28, although at the end of the intervention there was no difference between groups and a similar trend was observed for postprandial glucose. | 3 |
| Netherlands  2017 | Male and female.  45 – 70 years old.  Prediabetic. | 44 | **Intervention:** Ingested 15 g GOS daily with their regular meals.  **Control:** Isocaloric placebo (maltodextrin) daily with their regular meals. | 12 weeks | **Primary:** This study was to determine the long-term effects of supplementation with GOS, an acetogenic fiber, on the composition of the human gut microbiota and human metabolism. | Supplementation of diets with GOS, but not placebo, increased the abundance of *Bifidobacterium* species in feces by 5-fold (*P* =0.009; q =0.144). Microbial richness or diversity in fecal samples was not affected. | Peripheral insulin sensitivity as assessed by the M-value was not changed after GOS treatment as compared with placebo (P=0.467). The Homeostasis Model (HOMA-IR) did not differ between treatments (P=0.598). In addition, insulin-stimulated FFA suppression, a measure for adipose tissue insulin sensitivity, was not affected by GOS as compared to placebo (P=0.808). | 4 |
| Malaysia  2017 | Male and female.  30 – 70 years old.  BMI between 18.5 - 40 kg/m^2^.  T2D. | 136 | **Intervention:** Standard diet and probiotic sachets.  **Control:** Standard diet and placebo. | 12 weeks | **Primary:** This study was to determine the effect of multi-strain microbial cell preparation-also refers to multi-strain probiotics on glycemic control and other diabetes-related outcomes in people with type 2 diabetes. | The CFUs of *Bifidobacterium* spp. increased marginally (almost twofold) in the placebo group, the CFUs have increased significantly (up to 4.5-fold) in the probiotic group. The quantities of *Lactobacillus* spp increased in both groups. | The HbA1c has marginally increased in placebo group, while it decreased 0.14 ± 0.41 % in probiotics group. These changes were significant between groups (p < 0.05, effect size 0.050), but not significantly different within each group. According to within-subject contrast test, these changes were significant between baseline and week 6 and between baseline and week 12 (p < 0.05). ITT analysis revealed that HbA1c remained unchanged in the placebo group while marginally decreased in the probiotics group, although not to a statistically significant extent. | 5 |
| Italy  2016 | Male and female.  40 – 77 years old.  BMI 27–45 kg/m^2^.  T2D | 34 | **Intervention:** The Ma-Pi 2 diet.  **Control:** A diet based on the dietary guidelines for T2D recommended by professional societies in Italy. | 21 days | **Primary:** This study was to determine the possibility of improving metabolic control in T2D by correcting gut microbiome dysbioses through diet has been evaluated. | Both diets modulate the dysbiosis of the intestinal microbiome in type 2 diabetes, increasing the bacteria that produce SCFA (*Faecalibacterium*, *Roseburia*, *Lachnospira*, *Bacteroides*, *and Akkermansia*). The Ma-Pi 2 diet increased pro-inflammatory groups (*Collinsella* and *Streptococcus*), showing the potential to reverse pro-inflammatory dysbiosis. | The Ma-Pi 2 diet group showed a significantly higher reduction in HOMA-IR compared with the CTR group. Furthermore, although both diets were effective in reducing the plasma TNF-α levels, only the Ma-Pi 2 dietary intervention resulted in a significant reduction in plasma levels of CRP and IL-6. | 6 |
| UK  2016 | Male with well-controlled T2D.  42 - 65 years old. | 29 | **Intervention:** Prebiotic (galacto-oligosaccharide mixture) 5.5g/d.  **Control:** Placebo (maltodextrin) supplement 5.5 g/d. | 12 weeks | **Primary:** This study compared the effects of prebiotic supplementation with placebo treatment for 12 weeks on glucose control, IP, intestinal bacterial composition, endotoxemia, and inflammatory markers in patients with T2D. | Prebiotic fiber treatment did not induce significant changes in diversity, evenness, and richness indices when compared with placebo. However, bacterial diversity, as assessed by the Shannon and inverse Simpson indices, and richness increased significantly within the prebiotic group.  Prebiotic treatment had no significant effect on *Bifidobacterium* or any of the other bacteria measured. *Bifidobacterium* levels increased in both groups; however, the change within the prebiotic group was greater and close to significance (*P*=0.0582). | Prebiotic treatment had no significant effect on glucose, insulin and C-peptide fasting concentrations or responses during OGTT compared with placebo. The change in glucose effectiveness at zero insulin in the placebo group was significantly different from the prebiotic group. There were no significant effects of prebiotic treatment on inflammatory markers. | 7 |
| Spain  2016 | Male and female.  40 – 70 years old.  BMI between 26 - 35 kg/m^2^ T2D. | 35 | **Intervention:** Standard diet enriched with 100 g of sardines 5 days a week.  **Control:** Standard diet. | 6 months | **Primary:** This pilot study was to investigate the effects of a sardine-enriched diet on metabolic control, adiponectin, inflammatory markers, EMFA composition, and gut microbiota in drug-naïve patients with type 2 diabetes. | Both dietary interventions decreased phylum *Firmicutes* (SG and CG: *P* = 0.04) and increased *E. coli* concentrations (SG: *P* = 0.01, CG: *P* = 0.03) at the end of the study from baseline, whereas SG decreased *Firmicutes/Bacteroidetes* ratio (*P* = 0.04) and increased *Bacteroides-Prevotella* (*P* = 0.004) compared to baseline. | The changes in fasting glucose, HbA1c, fasting insulin and HOMA-IR values were similar and non-statistically different between the two intervention diets. Only CG decreased significantly Hba1c values compared to baseline (−0.3 % ± 0.1, P = 0.01). Both groups significantly reduced fasting insulin and HOMA-IR levels as compared with baseline values, and although the patients in the SG exhibited a greater decrease from baseline (SG: −6.1 ± 1.8 mU/L insulin, P = 0.01, −2.3 ± 0.7 HOMA-IR, P = 0.007, CG: −3.4 ± 1.5 mU/L insulin, P = 0.02, −1.1 ± 0.7 HOMA-IR, P = 0.04), the mean change from baseline to 6 months was not different between SG and CG. | 8 |
| Sweden  2016 | Male and female.  50 - 75 years old.  BMI between 25 - 45 kg/m^2^.  TD2. | 46 | **Intervention:** Low or High supplementation with *L. reuteri* DSM 17938.  **Control:** Placebo. | 12 weeks | **Primary:** This study was to investigate the metabolic effects of 12-week oral supplementation with *Lactobacillus reuteri* DSM 17938 in patients with type 2 diabetes on insulin therapy. | The participants who responded with increased ISI after *Lactobacillus reuteri* supplementation had higher microbial diversity at baseline, and increased serum levels of DCA after supplementation. | Supplementation with *L. reuteri* DSM 17938 for 12 weeks did not affect HbA1c. | 9 |
| India  2015 | Male and female.  35 – 55 years old. | 35 | **Intervention:** This group was supplemented with 1 g of freeze-dried synbiotic product (2 species of Lactobacillus, Bifidobacterium each, one species of Streptococcus, one species of yeast along with 300 mg Fructooligosaccharide) daily.  **Control:** no synbiotic supplementation. | 45 days | **Primary:** This study was to determine the effect of synbiotic supplementation on glycemia, gut health, and SCFA levels in prehypertensive type 2 diabetic adults. | Intervention with synbiotic supplementation resulted in a significant increment in butyrate (547.4%) and propionate (310%) levels. Gut health of the subjects improved significantly as indicated by increased colonization of *Bifidobacteria* (131.6%) and *Lactobacillus* (32.6%) and a significant reduction in enteric pathogens (44.6%). | Intervention with synbiotic supplementation resulted in a significant reduction in FBS, PP_2_BS, HbA1c, by 3.3%, 6.7%, 14%, respectively along with a significant increment in butyrate (547.4%) and propionate (310%) levels. | 10 |
| Korea  2013 | Male and female.  48 – 72 years old.  T2D. | 6 | **Intervention:** SVD. | 1 month | **Primary:** This study used diet therapy using an SVD to evaluate whether prebiotic consumption reduced the risk factors associated with metabolic diseases by  modulating the composition of the gut microbiota. | A strict vegetarian diet reduced the *Firmicutes*-to-*Bacteroidetes* ratio in the gut microbiota but did not alter enterotypes. There was a significant reduction in the concentrations of acetate and butyrate on day 28 compared with day 1 (P < 0.05). | An SVD reduced HbA1c, and improved fasting glucose and postprandial glucose levels. | 11 |
| Japan  2013 | Male and female.  50 - 75 years old.  T2D. | 60 | **Intervention:** TGD orally (300 or 900 mg/day).  **Control:** Placebo. | 12 weeks | **Primary:** This study was to evaluate the efficacy of TGD in modulating blood glucose levels and body weight gain in patients with type T2D.  **Secondary:** This study clarifies the underlying mechanism by analyzing the gut microbiota of T2D patients. | The *Clostridium* cluster IV and subcluster XIVa components were significantly decreased, whereas the *Lactobacillales* and *Bifidobacterium* populations significantly increased in the T2D patients. The *Bacteroidetes*-to-*Firmicutes* ratio in the TGD groups significantly increased and was significantly higher compared with that in the placebo group, indicating that TGD improved the growth of the fecal bacterial communities in the T2D patients. | In the placebo group, HbA1c levels were increased in 11 patients (69%) after the treatment; levels were higher but not significant than those in the TGD-treated group (14 [39%]; p = 0.07). In the placebo group. | 12 |

T2D: Diabetes Mellitus type 2; a-LCD: almond-based low carbohydrate diet; LFD: Low-fat diet; GLP-1: glucagon-like peptide 1; FDR: false discovery rate; BMI: body mass index; AA: amino acids; BCAA: branched-chain amino acids; SCFA: short-chain fatty acids; GOS: galactooligosaccharides; CFUs: colony-forming units; Ma-Pi 2: fiber-rich macrobiotic diet; DCA: deoxycholic acid; IP: intestinal permeability; EMFA: erythrocyte membrane fatty acid; SG: sardine group; CG: control group; SVD: Strict vegetarian diet; TGD: Transglucosidase; FBS: fasting blood glucose; PP2BS: postprandial blood glucose.

**Table S2** Search equations used with Scopus.

| **Equation 1** | TITLE-ABS-KEY ( "type 2 diabetes" ) OR TITLE-ABS-KEY ( "Gut microbiota" ) OR TITLE-ABS-KEY ( "gut microbiome" ) TITLE-ABS-KEY ( "Systems biology" ) AND TITLE-ABS-KEY ( "Bioinformatics" ) AND TITLE-ABS-KEY ( "Diet" ) AND ( LIMIT-TO ( DOCTYPE , "re" ) ) |
| --- | --- |
| **Equation 2** | TITLE-ABS-KEY ( "type 2 diabetes" ) OR TITLE-ABS-KEY ( "Gut microbiota" ) OR TITLE-ABS-KEY ( "gut microbiome" ) TITLE-ABS-KEY ( "Systems biology" ) AND TITLE-ABS-KEY ( "Bioinformatics" ) OR TITLE-ABS-KEY ( "Diet" ) OR TITLE-ABS-KEY ( "Lifestyle" ) AND ( LIMIT-TO ( DOCTYPE , "ar" ) OR LIMIT-TO ( DOCTYPE , "re" ) ) |

**Table S3**. Articles obtained with equation 1 (Table S2) in Scopus

| **Title** | **Authors** | **Year** | **Source** |
| --- | --- | --- | --- |
| Foodomics for human health: current status and perspectives | Braconi, D., Bernardini, G., Millucci, L., Santucci, A. | 2018 | Expert Review of Proteomics  15(2), pp. 153-164 |
| Linking Microbiota to Human Diseases: A Systems Biology Perspective | Wu, H., Tremaroli, V., Bäckhed, F. | 2015 | Trends in Endocrinology and Metabolism  26(12), pp. 758-770 |
| Potential value of nutrigenomics in Crohn's disease | Ferguson, L.R. | 2012 | Nature Reviews Gastroenterology and Hepatology 9(5), pp. 260-270 |
| Nutrition genomics \| [Nutriční genomika] | Šedová, L., Šeda, O., Sobotka, L. | 2004 | Casopis Lekaru Ceskych  143(10), pp. 676-679 |

**Table S4**. Articles obtained with equation 2 (Table S2) in Scopus.

| Title | Authors | Year | Source |
| --- | --- | --- | --- |
| Therapeutic endoscopy for the treatment of post-bariatric surgery complications  Open Access | Larsen, M., Kozarek, R. | 2022 | World Journal of Gastroenterology  28(2), pp. 199-215 |
| Understanding the role of the gut microbiome and microbial metabolites in non-alcoholic fatty liver disease: Current evidence and perspectives  Open Access | Vallianou, N., Christodoulatos, G.S., Karampela, I., (...), Kounatidis, D., Dalamaga, M. | 2022 | Biomolecules  12(1),56 |
| Shifts in gut microbiota and their metabolites induced by bariatric surgery. Impact of factors shaping gut microbiota on bariatric surgery outcome | Gutiérrez-Repiso, C., Moreno-Indias, I., Tinahones, F.J. | 2021 | Reviews in Endocrine and Metabolic Disorders  22(4), pp. 1137-1156 |
| Molecular Aspects of Lifestyle and Environmental Effects in Patients With Diabetes: JACC Focus Seminar | Nayor, M., Shah, S.H., Murthy, V., Shah, R.V. | 2021 | Journal of the American College of Cardiology  78(5), pp. 481-495 |
| Nonalcoholic fatty liver disease (Nafld) as model of gut–liver axis interaction: From pathophysiology to potential target of treatment for personalized therapy  Open Access | Fianchi, F., Liguori, A., Gasbarrini, A., Grieco, A., Miele, L. | 2021 | International Journal of Molecular Sciences  22(12),6485 |
| Reducing Kidney Function Decline in Patients With CKD: Core Curriculum 2021  Open Access | Chen, T.K., Sperati, C.J., Thavarajah, S., Grams, M.E. | 2021 | American Journal of Kidney Diseases  77(6), pp. 969-983 |
| Splenic Abscess Following Sleeve Gastrectomy: A Systematic Review of Clinical Presentation and Management Methods  Open Access | Sakran, N., Zakeri, R., Madhok, B., (...), Shah, K., Pouwels, S. | 2021 | Obesity Surgery  31(6), pp. 2753-2761 |
| Mining Gut Microbiota From Bariatric Surgery for MAFLD  Open Access | Wu, W.-K., Chen, Y.-H., Lee, P.-C., (...), Liu, C.-J., Wu, M.-S. | 2021 | Frontiers in Endocrinology  12,612946 |
| What’s in a Name? Redefining Type 2 Diabetes Remission  Open Access | Kalra, S., Singal, A., Lathia, T. | 2021 | Diabetes Therapy  12(3), pp. 647-654 |
| Microbiome: Role and functionality in human nutrition cycle  Open Access | Alhusain, F. | 2021 | Saudi Medical Journal  42(2), pp. 146-150 |
| Gut microbiota: a target for intervention in obesity | Sehgal, K., Khanna, S. | 2021 | Expert Review of Gastroenterology and Hepatology  15(10), pp. 1169-1179 |
| Bariatric procedures and microbiota: patient selection and outcome prediction  Open Access | Puca, P., Petito, V., Laterza, L., (...), Gasbarrini, A., Scaldaferri, F. | 2021 | Therapeutic Advances in Gastrointestinal Endoscopy 14 |
| Obesity in pregnancy: A new chapter in obstetrics  Open Access | Antsaklis, A. | 2021 | Donald School Journal of Ultrasound in Obstetrics and Gynecology  15(1), pp. 43-48 |
| Brain–gut–microbiome interactions in obesity and food addiction  Open Access | Gupta, A., Osadchiy, V., Mayer, E.A. | 2020 | Nature Reviews Gastroenterology and Hepatology  17(11), pp. 655-672 |
| The role of the gut microbiome and diet in the pathogenesis of non-alcoholic fatty liver disease  Open Access | Jennison, E., Byrne, C.D | 2020 | Clinical and Molecular Hepatology  27(1), pp. 22-43 |
| Metabolic networks of the human gut microbiota  Open Access | Selber-Hnatiw, S., Sultana, T., Tse, W., (...), Zouboulakis, K., Gamberi, C. | 2020 | Microbiology (United Kingdom)  166(2), pp. 96-119 |
| Gut microbiota and obesity: A role for probiotics  Open Access | Abenavoli, L., Scarpellini, E., Colica, C., (...), Izzo, A.A., Capasso, R. | 2019 | Nutrients  11(11),2690 |
| Hypertension as a Metabolic Disorder and the Novel Role of the Gut  Open Access | Tanaka, M., Itoh, H. | 2019 | Current Hypertension Reports  21(8),63 |
| Obesity, diabetes, and the gut microbiome: an updated review | Singer-Englar, T., Barlow, G., Mathur, R. | 2019 | Expert Review of Gastroenterology and Hepatology  13(1), pp. 3-15 |
| All disease begins in the gut: Influence of gastrointestinal disorders and surgery on oral drug performance  Open Access | Hatton, G.B., Madla, C.M., Rabbie, S.C., Basit, A.W. | 2018 | International Journal of Pharmaceutics  548(1), pp. 408-422 |
| Gut microbiota and body weight – a review \| [Microbioma intestinal y peso corporal – revisión]  Open Access | Duca, I., Rusu, F., Chira, A., Dumitrascu, D.L. | 2018 | Psihologijske Teme  27(1), pp. 33-53 |
| Evaluating causality of gut microbiota in obesity and diabetes in humans  Open Access | Meijnikman, A.S., Gerdes, V.E., Nieuwdorp, M., Herrema, H. | 2018 | Endocrine Reviews  39(2), pp. 133-153 |
| Reciprocal interactions between bile acids and gut microbiota in human liver diseases | Ikegami, T., Honda, A. | 2018 | Hepatology Research  48(1), pp. 15-27 |
| The Microbiome That Shapes Us: Can It Cause Obesity? . | Omer, E., Atassi, H | 2017 | Current Gastroenterology Reports  19(12),59 |
| Strategies to increase the efficacy of using gut microbiota for the modulation of obesity | Li, J., Riaz Rajoka, M.S., Shao, D., (...), Yang, H., Shi, J. | 2017 | Obesity Reviews  18(11), pp. 1260-1271 |
| Personalized microbiome-based approaches to metabolic syndrome management and prevention  Open Access | Shapiro, H., Suez, J., Elinav, E. | 2017 | Journal of Diabetes  9(3), pp. 226-236 |
| Beyond gut microbiota: Understanding obesity and type 2 diabetes  Open Access | Lau, E., Carvalho, D., Pina-Vaz, C., Barbosa, J.-A., Freitas, P. | 2015 | Hormones  14(3), pp. 358-369 |
| The gut microbiome in cardio-metabolic health  Open Access | Hansen, T.H., Gøbel, R.J., Hansen, T., Pedersen, O. | 2015 | Genome Medicine  7(1),33 |
| Gut microbiota and metabolic syndrome  Open Access | Festi, D., Schiumerini, R., Eusebi, L.H., (...), Taddia, M., Colecchia, A. | 2014 | World Journal of Gastroenterology  20(43), pp. 16079-16094 |
| Gut microbiota and obesity: Role in aetiology and potential therapeutic target | Moran, C.P., Shanahan, F. | 2014 | Best Practice and Research: Clinical Gastroenterology  28(4), pp. 585-597 |
| The human microbiome and bile acid metabolism: Dysbiosis, dysmetabolism, disease and intervention | Jones, M.L., Martoni, C.J., Ganopolsky, J.G., Labbé, A., Prakash, S. | 2014 | Expert Opinion on Biological Therapy  14(4), pp. 467-482 |
| The gordian knot of dysbiosis, obesity and nafld . | Mehal, W.Z | 2013 | Nature Reviews Gastroenterology and Hepatology  10(11), pp. 637-644 |
| Influence of Gut microbiota on subclinical inflammation and insulin resistance  Open Access | Carvalho, B.M., Abdalla Saad, M.J. | 2013 | Mediators of Inflammation  2013,986734 |
| Bridging immunity and lipid metabolism by gut microbiota | Greer, R.L., Morgun, A., Shulzhenko, N. | 2013 | Journal of Allergy and Clinical Immunology  132(2), pp. 253-262 |
| The importance of the gut microbiota after bariatric surgery | Aron-Wisnewsky, J., Doré, J., Clement, K. | 2012 | Nature Reviews Gastroenterology and Hepatology  9(10), pp. 590-598 |
| Do interactions between gut ecology and environmental chemicals contribute to obesity and diabetes?  Open Access | Snedeker, S.M., Hay, A.G. | 2012 | Environmental Health Perspectives  120(3), pp. 332-339 |
| Sleeve Gastrectomy: Procedure, Outcomes, and Complications  Open Access | Gill, R.S., Lai, M., Birch, D.W., Karmali, S. | 2012 | Current Obesity Reports  1(2), pp. 75-79 |
| The therapeutic potential of manipulating gut microbiota in obesity and type 2 diabetes mellitus | Kootte, R.S., Vrieze, A., Holleman, F., (...), Stroes, E.S., Nieuwdorp, M. | 2012 | Diabetes, Obesity and Metabolism  14(2), pp. 112-120 |
| Use of probiotics as prophylaxis for postoperative infections  Open Access | Jeppsson, B., Mangell, P., Thorlacius, H. | 2011 | Nutrients  3(5), pp. 604-612 |


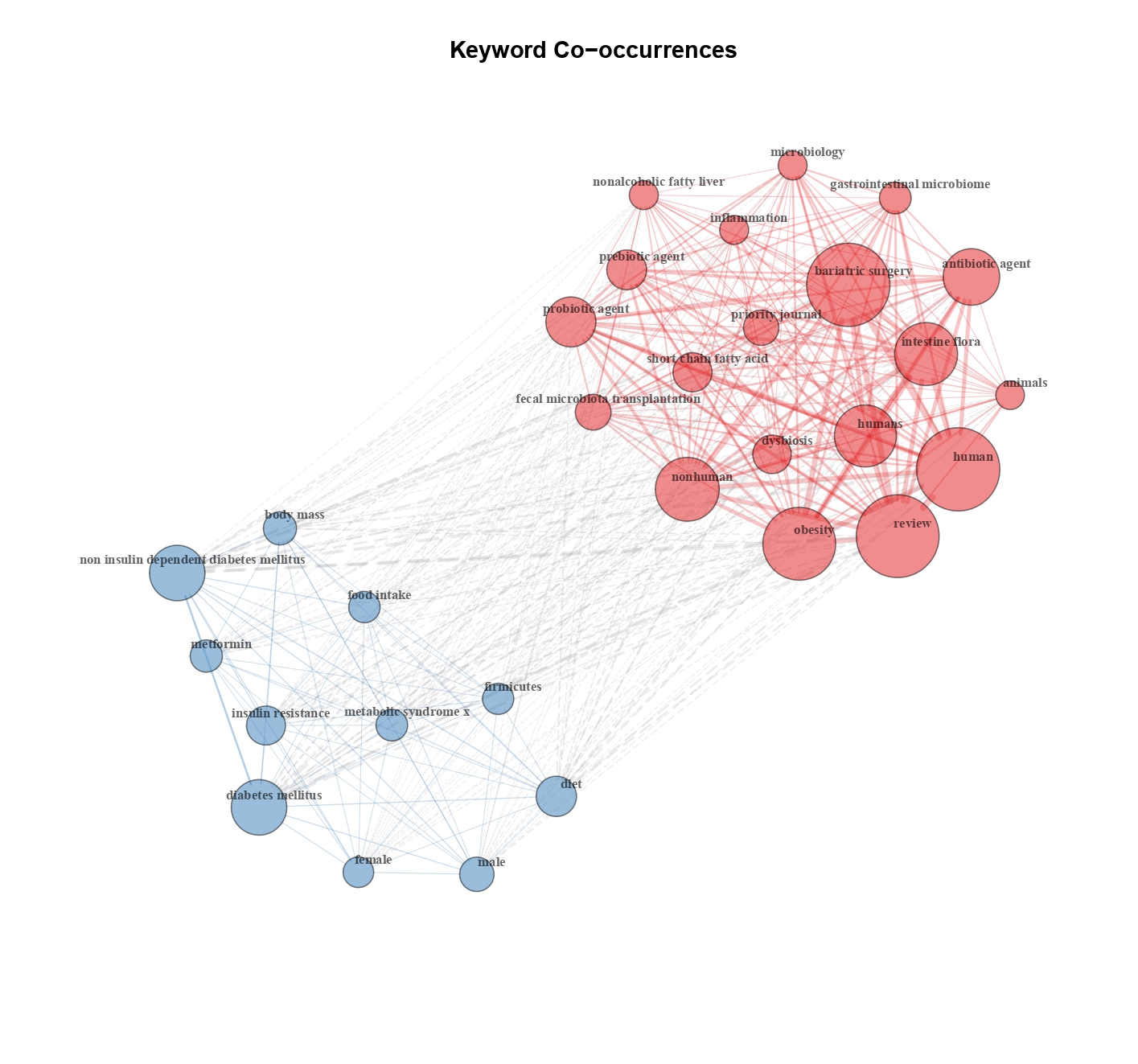


**Figure S1.** Keyword Co-occurrences network. The colors (red and blue) represent the conceptual groups based on keywords relationship. Size of the nodes are related to the keyword count in our Scopus directed search. Edges correspond to the interactions between nodes.


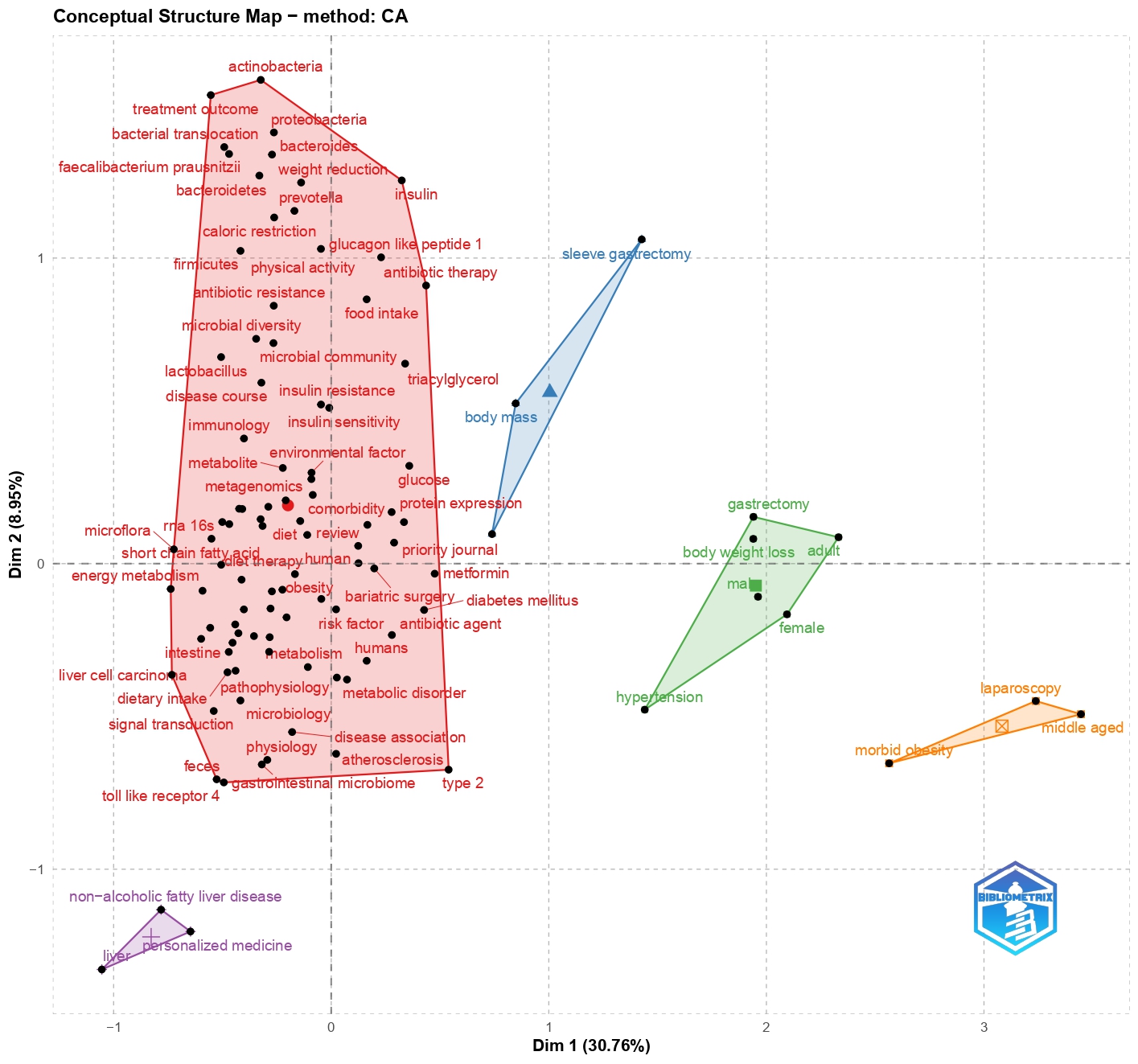


**Fig S2**. Conceptual structure map calculated by correspondence analysis (CA). CA allows to draw a conceptual structure of the field and detect clusters of documents which express common concepts. The colors represent the conceptual clusters detected through CA.


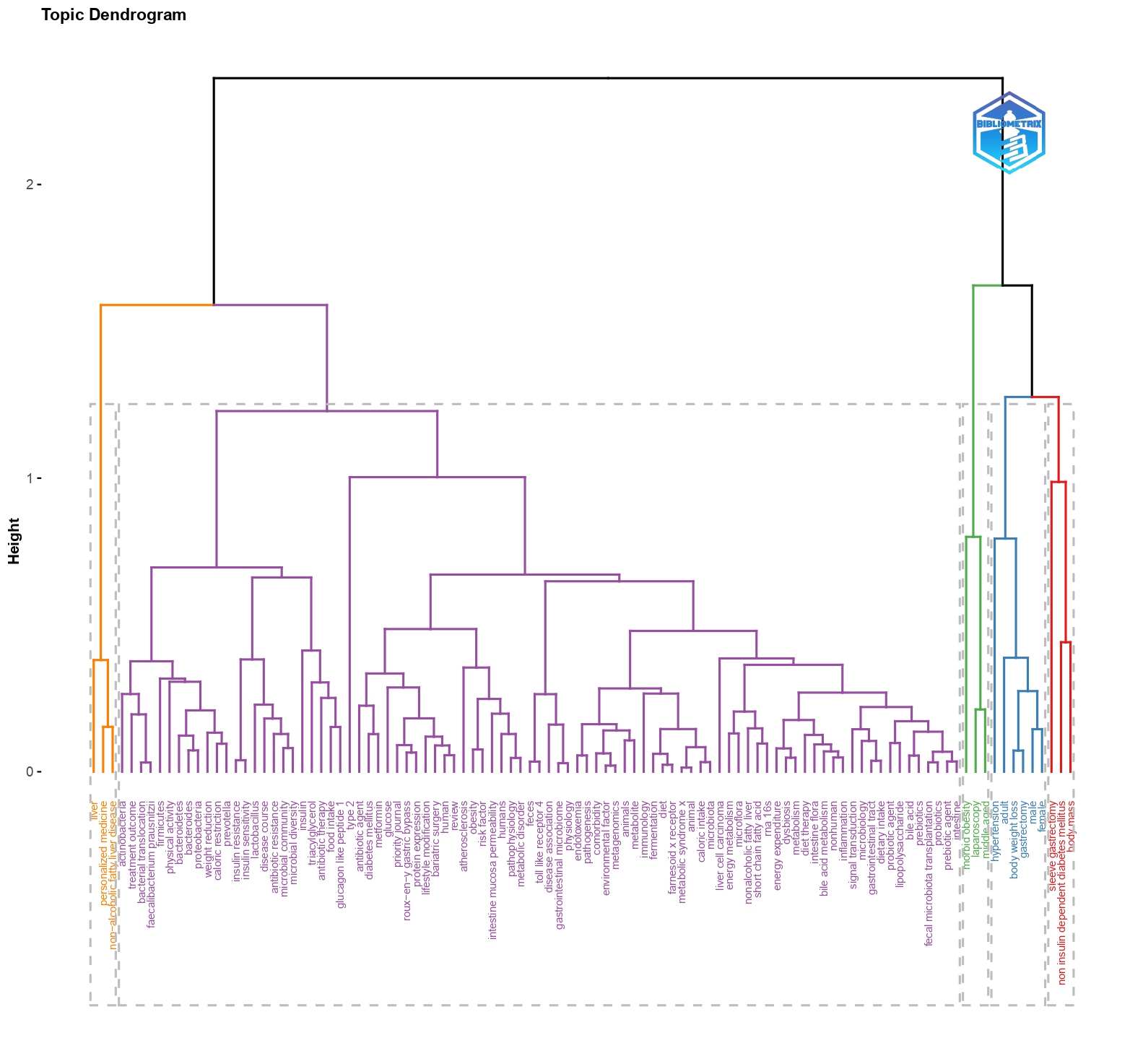


**Fig S3.** Topic dendrogram. The colors represent the conceptual clusters collapsed by topics.

References

1. Kanazawa A, Aida M, Yoshida Y, Kaga H, Katahira T, Suzuki L, et al. Effects of Synbiotic Supplementation on Chronic Inflammation and the Gut Microbiota in Obese Patients with Type 2 Diabetes Mellitus: A Randomized Controlled Study. Nutrients 2021; 13:558.

2. Karusheva Y, Koessler T, Strassburger K, Markgraf D, Mastrototaro L, Jelenik T, et al. Short-term dietary reduction of branched-chain amino acids reduces meal-induced insulin secretion and modifies microbiome composition in type 2 diabetes: a randomized controlled crossover trial. The American journal of clinical nutrition 2019; 110:1098-107.

3. Zhao L, Zhang F, Ding X, Wu G, Lam YY, Wang X, et al. Gut bacteria selectively promoted by dietary fibers alleviate type 2 diabetes. Science 2018; 359:1151-6.

4. Canfora EE, van der Beek CM, Hermes GD, Goossens GH, Jocken JW, Holst JJ, et al. Supplementation of diet with galacto-oligosaccharides increases bifidobacteria, but not insulin sensitivity, in obese prediabetic individuals. Gastroenterology 2017; 153:87-97. e3.

5. Firouzi S, Majid HA, Ismail A, Kamaruddin NA, Barakatun-Nisak M-Y. Effect of multi-strain probiotics (multi-strain microbial cell preparation) on glycemic control and other diabetes-related outcomes in people with type 2 diabetes: a randomized controlled trial. European journal of nutrition 2017; 56:1535-50.

6. Candela M, Biagi E, Soverini M, Consolandi C, Quercia S, Severgnini M, et al. Modulation of gut microbiota dysbioses in type 2 diabetic patients by macrobiotic Ma-Pi 2 diet. British Journal of Nutrition 2016; 116:80-93.

7. Pedersen C, Gallagher E, Horton F, Ellis RJ, Ijaz UZ, Wu H, et al. Host–microbiome interactions in human type 2 diabetes following prebiotic fibre (galacto-oligosaccharide) intake. British Journal of Nutrition 2016; 116:1869-77.

8. Balfegó M, Canivell S, Hanzu FA, Sala-Vila A, Martínez-Medina M, Murillo S, et al. Effects of sardine-enriched diet on metabolic control, inflammation and gut microbiota in drug-naïve patients with type 2 diabetes: a pilot randomized trial. Lipids in health and disease 2016; 15:1-11.

9. Mobini R, Tremaroli V, Ståhlman M, Karlsson F, Levin M, Ljungberg M, et al. Metabolic effects of L actobacillus reuteri DSM 17938 in people with type 2 diabetes: A randomized controlled trial. Diabetes, Obesity and Metabolism 2017; 19:579-89.

10. Sheth M, Chand V, Thakuria A. Inflated levels of SCFA, Bifidobacteria and Lactobacillus improves the status of pre hypertension and type 2 diabetes mellitus in subjects residing in north east India—a randomized control trial with synbiotic supplementation. Int J Curr Pharm Res 2015; 7:33-6.

11. Kim MS, Hwang SS, Park EJ, Bae JW. Strict vegetarian diet improves the risk factors associated with metabolic diseases by modulating gut microbiota and reducing intestinal inflammation. Environmental microbiology reports 2013; 5:765-75.

12. Sasaki M, Ogasawara N, Funaki Y, Mizuno M, Iida A, Goto C, et al. Transglucosidase improves the gut microbiota profile of type 2 diabetes mellitus patients: a randomized double-blind, placebo-controlled study. BMC gastroenterology 2013; 13:1-7.
